# Optoelectronic‐Driven van der Waals Ferroelectric Materials‐Based Memory Devices for Retinomorphic and In‐Sensory Hardware

**DOI:** 10.1002/advs.74794

**Published:** 2026-03-14

**Authors:** Parthasarathi Pal, Yeong‐Her Wang, Sanjay Kumar, Themis Prodromakis

**Affiliations:** ^1^ Department of Electrical Engineering Indian Institute of Technology Patna Bihar India; ^2^ Department of Electrical Engineering National Cheng Kung University Tainan Taiwan; ^3^ Centre For Electronics Frontiers School of Engineering The University of Edinburgh Scotland UK

**Keywords:** coupling polarization, ferroelectric materials, in‐memory computation, in‐sensory system, optoelectronic memories, switching dynamics

## Abstract

2D ferroelectric materials have recently emerged as a promising class of atomically thin semiconductors capable of integrating sensing, memory, and computation within a single device. Their unique combination of spontaneous switchable polarization, strong light‐matter coupling, and van der Waals (vdW) interface compatibility provides an ideal platform for next‐generation optoelectronic vision sensors. Coupling ferroelectric polarization with photoresponse, 2D ferroelectric materials such as *α*‐In_2_Se_3_, CuInP_2_S_6_ (CIPS), SnS, and WTe_3_ enable non‐volatile modulation of photocarrier transport, facilitating adaptive visual perception analogous to the human retina. These 2D ferroelectric photonic devices demonstrate synaptic plasticity, short‐term and long‐term memory, and optical potentiation and depression characteristics under visible and near‐infrared excitation. Integrating ferroelectricity into optoelectronic architectures addresses the von‐Neumann bottleneck by enabling in‐sensor computing, where data are sensed, stored, and processed locally, minimizing latency and energy consumption. This review provides a comprehensive overview of 2D ferroelectric materials and their device architectures in the memristive and memtransistors devices structures for optoelectronic vision sensors, highlighting their polarization mechanism, light‐driven conductance modulation, and neuromorphic functionalities. Additionally, current challenges, such as scalability, polarization fatigue, and interface engineering, have also been extensively discussed together with heterostructure design and hybrid ferroelectric‐semiconductor integration toward energy‐efficient bio‐inspired vision systems.

## Graphical abstract

2D ferroelectrics materials enabling non‐volatile polarization memory, optical excitability, and neuromorphic processing within a unified material and provides a mechanistic analysis of polarization‐induced band modulation, including photon‐assisted domain reorientation, switching kinetics, and interfacial dipole coupling that governs resistive switching and optoelectronic synaptic in 2D ferroelectrics. 2D ferroelectric memristors, FeFETs, and Fe‐Memtransistors are emphasized on light‐tunable multilevel state, ultralow switching energies, and neuromorphic visual capabilities.

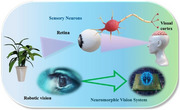

## 1 Introduction

The rapid expansion of data‐intensive applications in artificial intelligence (AI), edge computing, and interactive robotics has fueled intense research into hardware architectures capable of emulating biological perception and cognition. Conventional von Neumann architectures suffer from a fundamental bottleneck that arises from the physical separation between sensing, memory, and computation units, resulting in excessive latency and power dissipation during data transfer [1, 2, 3, 4, 5, 6, 7]. To overcome these constraints, in‐sensor and in‐memory computing paradigms have been introduced, wherein sensing and computation occur within the same physical domain [8, 9, 10]. Among emerging materials systems, 2D ferroelectrics have attracted exceptional attention as enablers of such architectures due to their switchable spontaneous polarization, atomic‐scale thickness, and strong coupling to optical and electrical stimuli [11, 12].

Since the discovery of ferroelectricity [13] in vdW layered crystals such as *α*‐indium(III) selenide (*α*‐In_2_Se_3_), copper indium thiophosphate (CuInP_2_S_6_ or CIPS), tin sulfide (SnS), and tungsten tritelluride (WTe_3_) 2D ferroelectrics have redefined the physical limits of polarization stability at the nanoscale [14, 15, 16, 17, 18, 19, 20]. Unlike conventional perovskite ferroelectrics that lose polarization below a critical thickness because of depolarization fields, 2D ferroelectrics maintain robust dipole alignment even in the monolayer limit, owing to their dangling bond‐free surface and weak interlayer coupling [21, 22, 23, 24]. Their intrinsic semiconducting or metallic nature further allows seamless integration with optoelectronic circuits, creating opportunities for devices that can sense, store, and process optical information concurrently [25, 26, 27].

In parallel, 2D transition‐metal‐dichalcogenides (2D TMDCs), such as Molybdenum Disulfide (MoS_2_), tungsten disulfide (WS_2_), molybdenum ditelluride (MoTe_2_), and Rhenium disulfide (ReS_2_), have demonstrated remarkable light‐matter interactions, including broadband photoabsorption and tunable excitonic responses [28, 29, 30, 31, 32]. Combining ferroelectric polarization with these optoelectronic 2D materials yields hybrid heterostructures capable of non‐volatile optical modulation and memory retention. Ferroelectric gating in MoS_2_ and WS_2_ channels has enabled persistent photoconductivity and optical plasticity behaviors analogous to synaptic potentiation and depression observed in biological vision [33, 34]. Such ferroelectric‐optoelectronic synapses bridge the gap between photodetection and neuromorphic processing, paving the way for bioinspired vision sensors with self‐adaptive learning functionalities [11, 35].

At the core of these systems lies the ferroelectric polarization switching mechanism, which modulates carrier concentration, barrier height, and recombination dynamics in the photoactive channel [36, 37]. Depending on the crystal symmetry, 2D ferroelectrics can exhibit in‐plane, out‐of‐plane, and interlocked polarization. Materials, such as tin telluride (SnTe) and SnS, exhibit in‐plane polarization resistant to depolarization fields [38, 39, 40], whereas *α*‐In_2_Se_3_ exhibits coupled in‐plane and out‐of‐plane dipoles, enhancing charge separation efficiency [41]. CIPS, with its off‐center Cu‐ion ordering, provides stable out‐of‐plane polarization suitable for vertical architectures. These structural anisotropies enable precise control of light‐induced conductance and memory behavior through polarization reversal and interfacial band alignment tuning [26].

**FIGURE 1:**
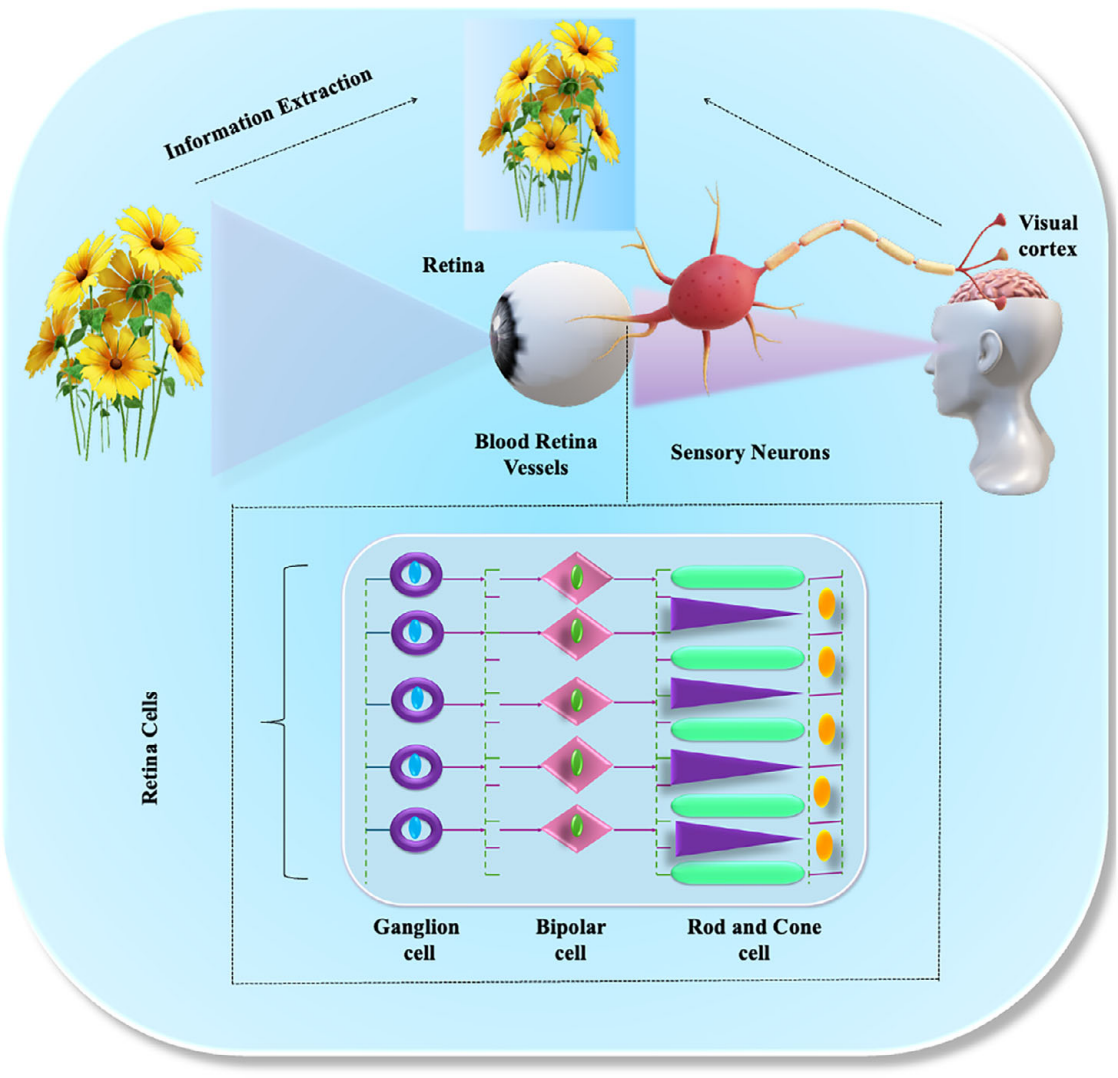
Schematic representation of the biological (human) vision system.

Optoelectronic vision sensors based on 2D ferroelectrics exploit the synergistic interplay of light excitation and ferroelectric polarization to emulate the spatiotemporal processing of retina [42] as shown in Figure 1. In such devices, optical pulses induce photogenerated carriers whose transport is modulated by the polarization state of the ferroelectric layer, producing excitatory post synaptic current (EPSC) and paired pulse facilitation (PPF) analogous to synaptic responses. Furthermore, the non‐volatility of ferroelectric domains allows the retention of optical stimuli as remanent conductance states, realizing long‐term potentiation (LTP) and long‐term depression (LTD) [43]. These behaviors collectively establish the foundation for neuromorphic vision perception, enabling pattern recognition and associative learning directly at the sensor level without external computation.

Recent demonstrations include *α*‐In_2_Se_3_ ferroelectric phototransistors exhibiting photo‐gated synaptic plasticity, and CIPS‐based heterojunctions showing polarization‐controlled photovoltage modulation, resulting in the dual‐optical‐electrical programming, combining photo‐induced carrier generation with polarization‐driven barrier tuning for efficient image recognition [42, 44, 45, 46]. These devices, integrated into crossbar or pixelated arrays, have enabled researchers to realize in‐sensor computing frameworks capable of parallel visual information processing and energy consumption at sub‐femtojoules per event [47, 48].

Nevertheless, several challenges remain before 2D ferroelectric materials can be widely adopted in practical vision sensors. First, the scalability and uniformity of large‐area 2D ferroelectric films need improvement through controllable synthesis methods such as chemical vapor deposition (CVD) or molecular beam epitaxy (MBE). The stability of polarization under repeated optical and electrical cycling must be enhanced to prevent fatigue or domain pinning. Also, precise band alignment engineering at the ferroelectric‐semiconductor interface is essential to suppress trap‐mediated recombination and to maintain high photoresponsivity [42, 43]. Finally, achieving 3D integration of 2D ferroelectric synaptic arrays with complementary–metal–oxide–semiconductor (CMOS) circuits will be critical for high‐resolution, energy‐efficient artificial vision hardware [49].

**FIGURE 2:**
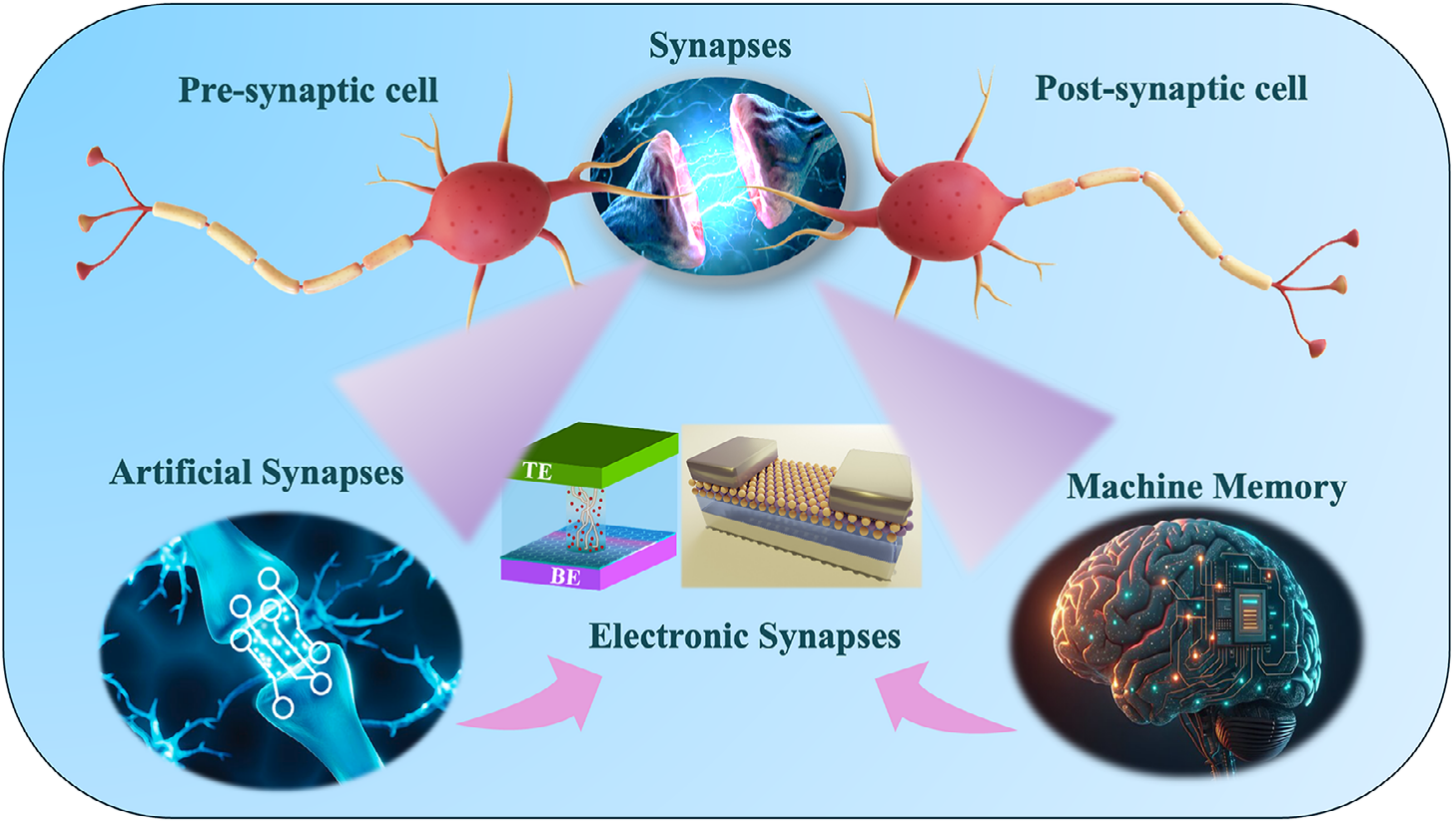
Schematic representation of non‐volatile memory‐based artificial synapses for in‐sensor computing.

2D ferroelectric materials represent a versatile and powerful platform for the realization of optoelectronic vision sensors that unify perception, memory, and computation. Their atomic thickness, switchable polarization, and compatibility with existing 2D semiconductors make them ideal for next‐generation neuromorphic and in‐sensor computing systems [42, 43, 49]. Continues advancements in material synthesis, interfacial control, and device design are expected to propel this field toward energy‐efficient, retina‐like visual systems capable of real‐time sensing and intelligent decision‐making at the hardware level, as depicted in Figure 2.

In this review, we summarize the latest progress in the integration of 2D ferroelectric materials and 2D materials, from the aspects of materials, device structure, working mechanism, and applications to neuromorphic sensing and computing. We have incorporated the experimental findings of the original mechanism of ferroelectricity in 2D ferroelectrics, depending on the direction of ferroelectric polarization. In the proceeding, two primary types of 2D ferroelectric structures are explained as per the different roles of ferroelectric components in the devices. Finally, the applications in the in‐memory computing (IMC) and in‐sensor computing systems of the 2D ferroelectric devices have been extensively discussed, and also the challenges and prospects in this rapidly developing research field have been summarized.

## 2 Materials Landscape of 2D Ferroelectrics

The discovery of the intrinsic ferroelectricity in atomically thin van der Waals (vdW) crystals has radically transformed the conventional understanding of polarization phenomena, which for decades were believed to be incompatible with the electrostatic and mechanical constraints of 2D systems [33, 34, 50]. The early observation of switchable polarization in CuInP_2_S_6_ (CIPS) and *α*‐In_2_Se_3_ demonstrated that the long‐range dipolar ordering can persist even when the material thickness is reduced to only a few atomic layers, thereby overturning the classical notion that depolarization fields can inevitably quench the ferroelectricity at the nanoscale [42]. Since then, a broad material landscape has emerged that includes not only layered chalcogenides but also group‐IV monochalcogenides, transition metal dichalcogenides exhibiting sliding ferroelectricity, and perovskite‐derived nanosheets. Together, these materials provide an unprecedented palette of physical mechanisms, starting from ionic displacement and order‐disorder transition to interlayer share coupling, through which stable, switchable polarization can be engineered in atomically thin form [49, 50, 51, 52, 53, 54].

### 2.1 Representative of 2D Ferroelectric Systems

Among the pioneering 2D ferroelectrics, CIPS remained the most thoroughly characterized. CIPS crystallizes in a layered monoclinic structure consisting of S‐P‐In‐Cu planes, weakly coupled by vdW forces. The Cu^+^ ions occupy off‐center octahedral sites within sulphur cages, producing a spontaneous out‐of‐plane dipole moment [55]. Its ferroelectric phase transition at approximately 320 K is of the order‐disorder type, arising from thermally activated Cu‐ion migration between metastable sites. Remarkably, piezo‐response‐force‐microscopy (PFM) studies have confirmed stable polarization switching down to 4 nm flakes, with a remnant polarization *P*_r_ ≈ 4 µC cm^−2^ and coercive force *E*_c_ ≈ 700 kV cm^−1^. The observation of long‐term domain retention exceeding 2 months, together with unusual phenomena, such as giant negative piezoelectricity and coexistence of ionic conduction with ferroelectric order, highlights the versatility of CIPS for device architectures that couple charge, ion, and lattice degrees of freedom [16, 42].

A second cornerstone material is *α*‐In_2_Se_3_, which exhibits both in‐plane and out‐of‐plane polarization components within a single quintuple‐layer unit (Se‐In‐Se‐In‐Se). The asymmetric stacking sequence (ABBCA) breaks inversion symmetry, yielding a non‐centrosymmetric lattice capable of dual‐axis polarization [18, 56]. First‐principles calculations predicted an extremely small switching barrier ∼0.06 eV per unit cell, enabling low‐voltage polarization reversal comparable to that of the classic perovskite ferroelectrics, such as lead titanate (PbTiO_3_) [43, 57]. Experimental confirmation of ferroelectricity down to monolayer limit (∼1.2 nm) was achieved through simultaneous second harmonic generation (SHG) and piezoresponse force microscopy (PFM) measurements, making *α*‐In_2_Se_3_ the thinnest intrinsic ferroelectric reported to date [51]. The closely related *α*‐In_2_Te_3_ shares structural motifs with *α*‐In_2_Se_3_ and has been predicted to possess out‐of‐plane polarization accompanied by strong piezoelectric coupling. Although its experimental exploration remains nascent, early studies indicate stable switchable domains and sizable polarization, extending the compositional tunability of group‐III chalcogenide ferroelectrics toward lower bandgap and enhanced infrared response [18, 49, 58, 59].

Recent advances in CVD grown large‐area 2D In_2_Se_3_ film transferred on the substrate to fabricate ferroelectric optoelectronic synapses demonstrated that switchable polarization could act as an intrinsic, non‐volatile ‘gate’ to modulate photoresponse, enabling a fundamentally different operating mechanism from conventional trap‐dominated photo‐memory. A vdW ferroelectric semiconductors were exploited to generate polarization‐controlled internal fields and domain configurations that regulate photocarrier separation, interfacial trapping and transport, thereby, producing multilevel optoelectronic plasticity with enhanced controllability and reduced reliance on stochastic defect states [60]. Complementarily, a synaptic platform established a polarization‐programmable optoelectronic synaptic platform in which ferroelectric electrostatics enable reconfigurable photogating and non‐volatile conductance modulation, allowing light‐driven learning behaviors and memory states within a single device structure [61]. Beyond single device demonstrations, a Ga_2_O_3_/*α*‐In_2_Se_3_ heterojunction ferroelectric optoelectronic sensor array translated the discussed mechanism, where polarization ‐modulated barrier engineering simultaneously suppresses dark current and amplifies solar‐blind UV detection, enabling pixel‐level artificial vision function, such as flame detection and motion perception [62].

The group‐IV monochalcogenides, such as SnS, tin selenide (SnSe), germanium monosulfide (GeS), germanium(II) selenide (GeSe) form another important family of 2D ferroelectrics, distinguished by their puckered orthorhombic lattices reminiscent of black phosphorus. The lack of minor plane along the armchair direction induces a spontaneous in‐plane polarization, which can be switched by uniaxial strain of external electric fields. Their polarization magnitude (1–4 µC cm^−2^) and moderate Curie temperatures (∼400 K) make them attractive for mechanically flexible and wearable ferroelectric devices. Moreover, the strong anisotropy of their electronic and optical properties supports polarization‐dependent carrier transportation and directional photoconduction, adding a further degree of control for anisotropic optoelectronic components [49, 51, 52, 53, 54].

Transition‐metal dichalcogenides (TMDCs) such as MoTe_2_, Tungsten ditelluride (WTe_2_) have recently been recognized as the hosts of sliding ferroelectricity, a mechanism distinct from conventional ionic displacement. In the rhombohedral bilayer MoTe_2_, relative interlayer translation breaks inversion symmetry and generates a reversible out‐of‐plane dipole [13, 21]. The vertical polarization can be switched by shear motion of the top layer under an in‐plane electric field, enabling ultrafast, fatigue‐resistant operation even beyond 10^6^ switching cycles [42]. In naturally parallel‐stacked multilayer ReSe_2_ (N ≥ 3), ferroelectricity originates from rational interlayer sliding of the middle layer, producing switchable polarization states that enable ferroelectric diode characteristics and programmable photovoltaic response with clear hysteretic signatures of polarization reversal [63]. Beyond electrical rectification, *α*‐In_2_Se_3_ exhibits even layer selective out‐of‐plane sliding ferroelectricity that induces a switchable bulk photovoltaic effect (BPVE) and ultrafast photocarrier dynamics down to ∼3 ps, supporting reconfigurable, self‐powered photodetection [64]. Importantly, 3R‐WS_2_ further exploits sliding‐enabled BPVE to realize nonvolatile responsivity reconfiguration +0.92 to −0.92 A W^−1^, surpassing p–n junction limits and enabling retinomorphic machine‐vision hardware [11]. This discovery generalizes ferroelectric behavior to centrosymmetric lattices and suggests that interlayer registry, rather than atomic displacement, can serve as the primary order parameter in 2D ferroelectrics [16, 65]. The extremely high Curie temperature (>650 K) and mechanical robustness of sliding ferroelectric TMDCs make them particularly promising for integrated memory and logic applications operating under harsh conditions [50, 66].

Finally, the ferroelectric perovskite nanosheets derived from layered oxides represent a complementary class of 2D‐like ferroelectrics [67]. When exfoliated to a few perovskite blocks, these nanosheets retain long‐range polarization while offering the chemical versatility of oxide systems [18, 66, 68]. However, their ferroelectricity tends to degrade rapidly below a few nanometers due to unscreened depolarization fields and interfacial strain, underscoring the unique advantage of vdW ferroelectrics, whose naturally passivated surface prevents charge compensation losses even in the monolayer limit [49, 68].

### 2.2 Polarization Switching Dynamics

The orientation and dynamics of polarization switching govern the functional design of 2D ferroelectric devices. In out‐of‐plane systems such as CIPS and *α*‐In_2_Se_3_, the vertical component of polarization directly modulates the channel potential in ferroelectric field effect transistors (FeFETs) or controls the tunneling barrier in ferroelectric tunnel junctions (FTJs) [69, 70]. Conversely, in‐plane ferroelectrics such as SnS and SnSe are naturally suited for lateral architectures, ferroelectric domain walls can act as conductive channels, and polarization reversal can be driven mechanically or by in‐plane fields, enabling low‐power, flexible device geometries.

**FIGURE 3:**
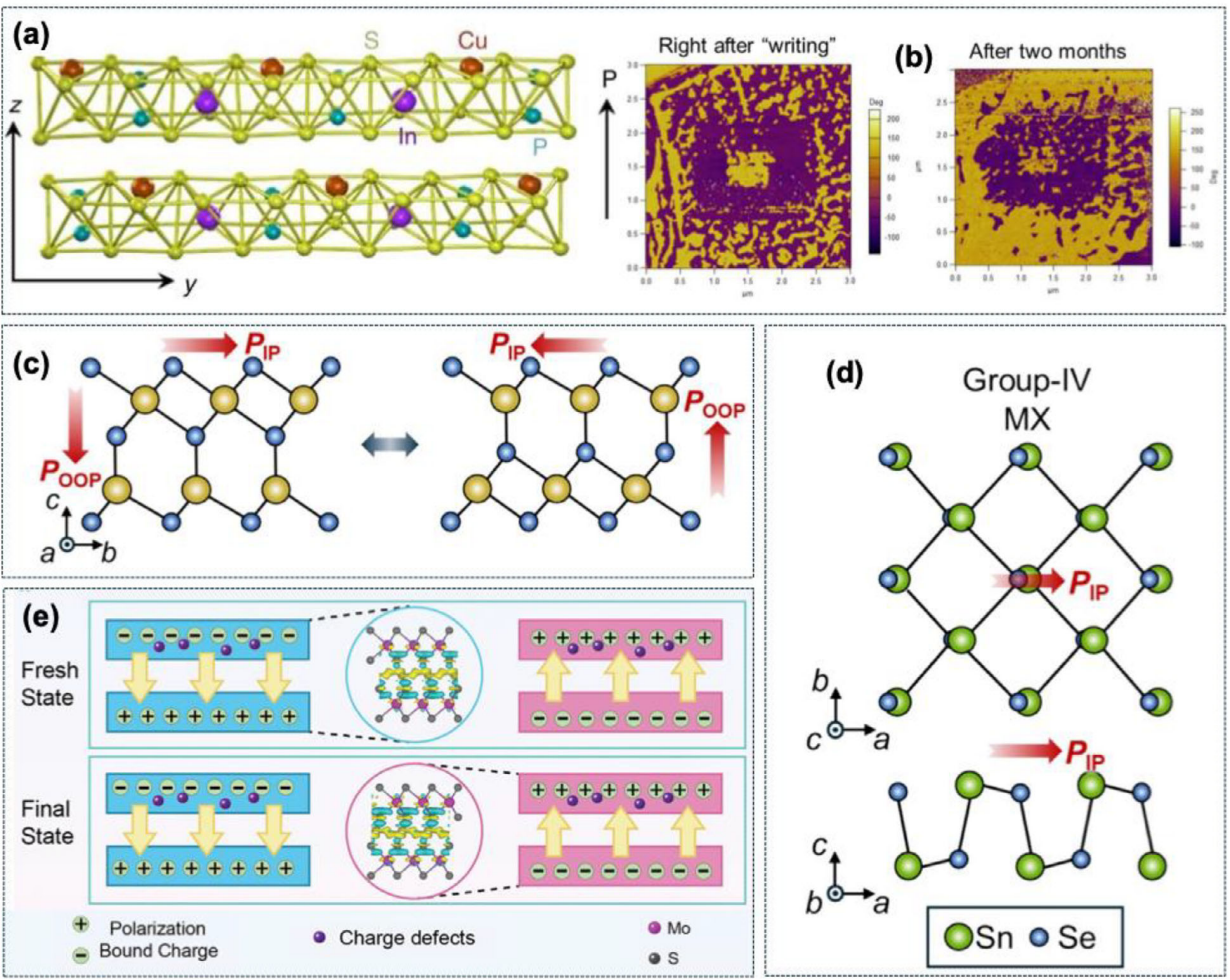
(a) The side view for the crystal structure of CIPS with a vdW gap between the layers. (b) Retention performance of the switched ferroelectric domains of a 30‐nm‐thick CIPS flake. (a) and (b) Reproduced with permission with Creative Commons CC BY [16]. 2026, Springer Nature. (c) Atomic configuration of α‐In_2_Se_3_. This material exhibits both in‐plane and out‐of‐plane polarization. (c) Reproduced with permission [56]. 2026, Elsevier. (d) Structure of multilayered SnSe with up and down ferroelectric polarization. Reproduced with permission with Creative Commons CC BY [16]. 2026, Springer Nature and with permission [56]. 2026, Elsevier (e) Schematic of sliding ferroelectric polarization‐switching mechanism based on the bilayer 3R‐MoS_2_ ferroelectrics. Reproduced with permission [32]. 2026, Science and reproduced with permission 49 2026, John Wiley and Sons.

Importantly, the interrelation of in‐plane and out‐of‐plane polarization in certain materials allows multi‐axis control. In *α*‐In_2_Se_3_, vertical electric fields induce synchronized reorientation of both components, offering bistable or even multi‐stable polarization states suitable for neuromorphic synapses and multilevel memories [38, 39]. The comparatively low coercive field of *α*‐In_2_Se_3_ (∼200 kV cm^−1^) relative to CIPS (∼700 kV cm^−1^) further facilitates low‐voltage operation, while the robust hysteresis behavior in sliding TMDC ferroelectrics ensures endurance without fatigue even under high frequency cycling [42]. The polarization dynamics in ferroelectric materials are shown in Figure 3.

### 2.3 Optical and Excitonic Coupling in 2D Ferroelectrics

Beyond purely electrical functionalities, the broken inversion symmetry of 2D ferroelectrics endows them with strong non‐linear optical responses and pronounced coupling between polarization and light absorption [11]. These materials exhibit a direct bandgap in the visible‐near‐infrared range (1–2.5 eV) and host tightly bound excitons due to reduced dielectric screening. The presence of spontaneous polarization breaks spatial inversion, giving rise to the bulk photovoltaic effect (BPVE) and other shift‐current phenomena that can exceed the Schockley‐Queisser limit of conventional p‐n junctions. In CIPS and *α*‐In_2_Se_3_, polarization reversal flips the direction of the photocurrent, providing an intrinsic mechanism for self‐powered photodetection and optical memory [71, 72, 73]. The ferroelectric control of exciton dissociation also leads to polarization‐tunable photoluminescence and enhanced photoresponsivity in hybrid heterostructures with graphene or TMDCs. Such polarization‐photon coupling lays the groundwork for retinomorphic computing, where optical sensing and memory coexist within the same ferroelectric device layer [42, 49].

### 2.4 Stability, Curie Temperature, and Fatigue Behavior

The retention of ferroelectric order at atomic thickness is one of the most striking advantages of vdW ferroelectrics over conventional oxides. Whereas perovskite thin films typically lose switchable polarization below ∼5 nm, vdW materials such as CIPS and *α*‐In_2_Se_3_ preserve ferroelectricity down to a single layer because their intrinsic vdW gaps effectively screen depolarizing fields and eliminate dangling bonds that would otherwise pin surface charges [42]. CIPS maintains a Curie temperature near 320 K, while *α*‐In_2_Se_3_ remains stable well above room temperature, sliding ferroelectric TMDCs even retain order up to 650 K. Nevertheless, extreme thickness reduction (< 3 nm) can lead to diminished coercive fields and local domain pinning, which may induce partial polarization fatigue under repeated cycling [16, 74, 75]. Protective encapsulation with inert 2D insulators such as hexagonal boron nitride (hBN) or integration into all‐vdW heterostructures (vdWH) has proven effective in suppressing environmental degradation and maintaining endurance beyond 10^6^ cycles [49, 76]. The combination of thermal robustness, mechanical flexibility, and chemical inertness renders 2D ferroelectrics uniquely suited for flexible and wearable electronics. Recent advances in 2D ferroelectric vdWH have established a new paradigm for highly integrated optoelectronic neuromorphic devices. Early demonstration of α‐In_2_Se_3_/GaSe heterostructure optoelectronic synapses validates polarization‐controlled synaptic plasticity to completely emulate Pavlov's learning in human brain for visual perception [37]. Building on this, two‐terminal full ferroelectric p–n vdWH neuristors achieved all‐in‐one visual perception with ultrahigh paired‐pulse facilitation (∼457%) and robust associative learning, enabled by reconfigurable polarization‐modulated built‐in fields for biological visual information perception [47]. A highly integrated polarization‐modulated optoelectronic synaptic transistor based on a 2D *α*‐In_2_Se_3_/SnS_2_ ferroelectric heterojunction demonstrates reversible ferroelectric polarization switching directly reprograms the interfacial barrier height and carrier distribution, enabling multimode optoelectronic functionality including reconfigurable AND/OR logic and optical wireless encoding via Morse‐code‐like photopulse sequences within a single compact architecture for neuromorphic machine vision hardware [77]. Their stability under ambient conditions contrasts sharply with the moisture‐sensitive perovskite oxides or organic ferroelectrics, extending the operational lifetime of 2D ferroelectric devices in a realistic environment [78, 79, 80].

Collectively, these materials delineate a broad design space for next‐generation ferroelectric nanodevices. As synthesis and transfer techniques mature, it is anticipated that 2D ferroelectrics will underpin an integrated platform for low‐power memories, logic‐in‐memory computing, and optoelectronic neuromorphic systems. Further study will likely focus on controlling domain topology, understanding interfacial coupling in vdW heterostructures, and exploiting the quantum mechanical origins of ferroelectricity at the monolayer limit.

### 2.5 Promising 2D Ferroelectrics for Optoelectronic Memory and Vision Systems

Recent advances in 2D ferroelectric materials have identified several material platforms as particularly promising for optoelectronic memory, neuromorphic computing, and bionic vision sensor applications. Among them *α*‐In_2_Se_3_ and CIPS have emerged as benchmark out‐of‐plane ferroelectric semiconductors, owing to their robust room‐temperature polarization, scalability to few‐layer thickness and compatibility with vdW heterostructures.

*α*‐In_2_Se_3_ offers a distinctive advantage by supporting coupled in‐plane and out‐of‐plane polarization, enabling efficient electrostatic modulation of carrier transport and polarization assisted separation of photogenerated carriers. These properties make *α*‐In_2_Se_3_ particularly attractive for integrated vision sensors that combine sensing, memory and computation within a single device structure [42]. Nevertheless, reported polarization strength, coercive field, and stability vary significantly across studies, highlighting unresolved challenges related to thickness‐dependent depolarization effects, interfacial screening and defect‐induced degradation.

CIPS exhibits stable switchable polarization down to the nanometer scale and has been widely implemented in FeFET, memristive devices, and optoelectronic synapses. However, its relatively large bandgap and order‐disorder ferroelectric nature may limit high speed optoelectronic operation and long‐term endurance under optical excitation [49].

Sliding ferroelectric materials and related vdW systems, constitute an emerging class of 2D ferroelectrics in which polarization originates from interlayer stacking asymmetry rather than ionic displacement [57]. These materials exhibit low switching energy, fast polarization dynamics and atomic‐scale thickness, making them attractive for low‐power neuromorphic vision hardware. However, practical deployment is currently limited by challenges in environmental stability, large‐area uniformity and precise control of interlayer sliding during fabrication. Ferroelectric polymers and ferro/2D semiconductor heterostructures also play an important supporting role by enabling polarization‐driven band modulation, enhanced photoresponse and NVM behavior [81]. While polymer ferroelectrics offer scalability and mechanical flexibility, their relatively slow switching speed and thermal stability remain concerns for dense neuromorphic integration.

Overall, existing studies indicate that no single 2D ferroelectric platform is universally optimal. Out‐of‐plane ferroelectrics such as *α*‐In_2_Se_3_, CIPS currently represent the most mature candidates for optoelectronic memory and retinal sensing, whereas sliding ferroelectrics hold strong long‐term potential for ultralow‐power neuromorphic architectures.

## 3 2D Ferroelectrics‐Based Bionic Vision Sensors

**FIGURE 4:**
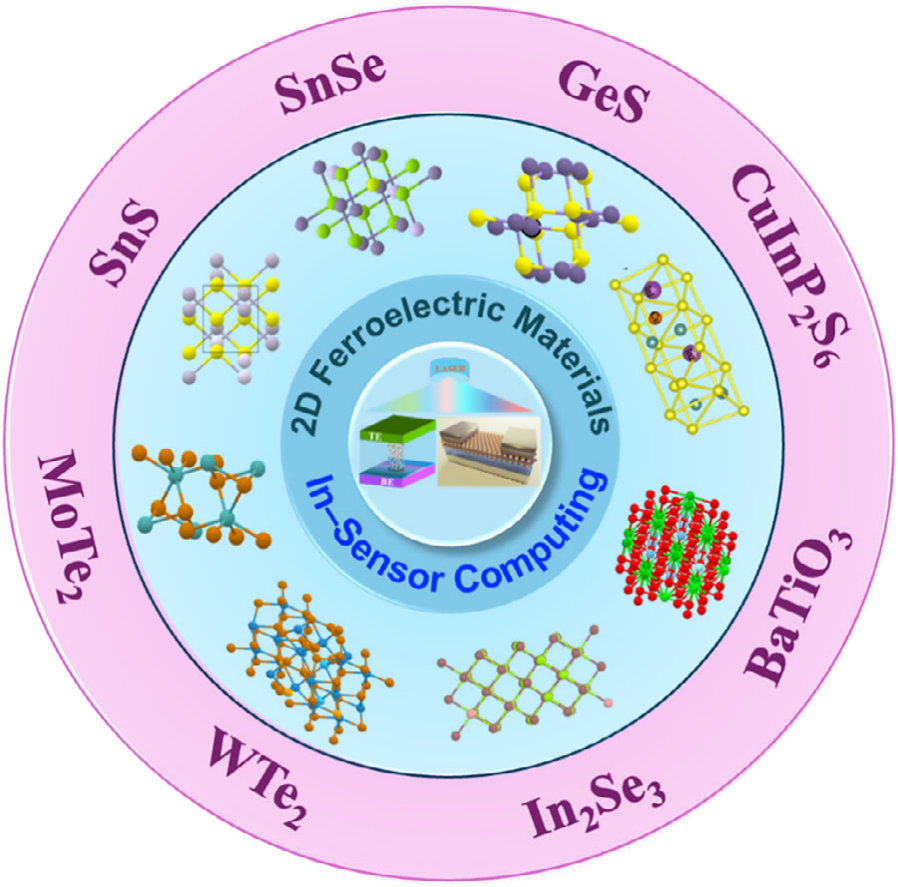
Materials landscape of 2D ferroelectrics for application in the non‐volatile memory‐based neuromorphic computing and in‐sensor computing.

The human retina performs visual preprocessing, such as detection, adaptation, and pattern encoding, directly at the sensory interface. Emulating such in‐sensor computing has become a central aim in neuromorphic optoelectronics, in which visual information is perceived and processed concurrently within a single pixel array. 2D ferroelectric materials shown in Figure 4 provide an elegant platform for realizing this bio‐inspired paradigm owing to their coexisting photoresponse, nonvolatile polarization, and atomic scale compatibility with vdW device integration [49, 65].

### 3.1 Device Engineering Strategies and Architectural Innovations

Building on the ferroelectric switching and polarization‐photocarrier coupling mechanisms discussed in previous section, recent progress in 2D ferroelectric optoelectronic vision hardware in increasingly defined by device engineering innovations rather than repeated mechanism exposition. In bionic vision system, the decisive metrices extend beyond photoresponsivity to include low‐leakage operation, multilevel programmability, compact pixel architectures, and array‐level uniformity, all of which demand deliberate heterointerface and structural design. Accordingly, state‐of‐the‐art demonstrations emphasize how ferroelectric polarization can be harnessed as a programmable electrostatic resource to reshape junction barriers, modulate built‐in fields and stabilize optoelectronic synaptic states under practical operating constraints. A dominant route is polarization‐programmable heterojunction design, where ferroelectric semiconductor functions as active electrostatic modulator to dynamically reshape band alignment and built‐in fields [62]. Beyond conventional heterojunctions, production‐driven progress also enabled by phase‐engineered ferroelectric heterophase junctions, in which polarization‐controlled characteristics is reinforced through phase boundary band alignment rather than relying solely on a homogeneous ferroelectric channel for an emerging platform for visual pattern memorization [60]. In parallel, the field is converging toward structurally simplified ferroelectric p–n heterojunction ultralow‐power photo‐programmability within compact geometry for sensory‐memory fusion at the pixel level [46]. More recently, sliding ferroelectric semiconductors have expanded the device design space toward ultrafast and low‐power sensory front‐ends for prospective reconfigurable, self‐powered, and high‐speed neuromorphic photodetection across broader 2D ferroelectric families [63, 64]. From a broader device‐engineering perspective, recent review analyses consistently conclude that most impactful advances in ferroelectric‐enhanced low‐dimensional optoelectronics are driven by heterointerface design, polarization‐assisted contact or barrier engineering, and leakage suppression, enabling simultaneous improvements in sensitivity, non‐volatility, and energy efficiency [81, 82, 83, 84]. Likewise, emerging 2D ferroelectric device roadmaps increasingly converge on in‐sensor and in‐memory computing architectures, where polarization programmability is translated into circuit compatible elements such as two‐terminal optoelectronic synapse and multi‐terminal ferroelectric photo‐transistors capable of multilevel conductance tuning. The architecture‐centric direction is particularly aligned with bionic vision requirements, as it enables spatiotemporal preprocessing and memory‐in‐pixel operation while mitigating the von‐Neumann data processing bottleneck [42, 49].

### 3.2 Bionic Functionalities and Neuromorphic Behavior

These 2D ferroelectric optoelectronic devices naturally reproduce several hallmarks of biological vision. First, adaptive gain control arises from the slow polarization or trap‐mediated relaxation processes, producing a logarithmic response to light intensity analogous to photoreceptor adaptation. Second, short‐term and long‐term plasticity manifest as transient or persistent conductance changes under optical pulse trains, which are vital for optical learning and visual memory. Third, spatially programmable polarization across a pixel array enables hardware convolution by encoding different responsivities (either positive or negative) into neighboring pixels; edge or motion detection can be achieved directly at the sensory front‐end without digital processing [67, 82, 85]. Finally, the intrinsic anisotropy of materials such as SnS or group‐IV monochalcogenides provides polarization‐selective and wavelength‐selective detection, extending sensory diversity similar to color and polarization vision in nature [42, 52].

### 3.3 Advantages and Remaining Challenges

Compared with oxide‐based ferroelectrics, 2D vdW ferroelectrics offer atomically sharp, defect‐free interfaces, ultralow switching voltages, and exceptional mechanical flexibility, which are essential for future wearable and vision systems [86, 87]. Their in‐plane integration through vdW stacking allows direct combination with other 2D semiconductors and transparent electrodes to realize dense, conformable sensor arrays [49]. Importantly, the strong light‐matter interaction and tunable band structure of these materials enhance quantum efficiency even in ultrathin geometries, permitting high responsivity at minimal energy cost.

Despite this promise, several challenges persist. The stability of ferroelectric polarization in ambient environments, particularly for Se‐based and Te‐based chalcogenides, remains a limiting factor. Repeated optical and electrical cycling can lead to partial depolarization or ionic migration, degrading device endurance. Moreover, reproducible large‐area synthesis of the ferroelectric phases (such as controlled synthesis of TMDCs or uniform CIPS films) is still under active development. Addressing these issues will be crucial for scalable integration of bionic vision sensor arrays [49, 50, 51, 52, 53, 88, 89, 90].

**FIGURE 5:**
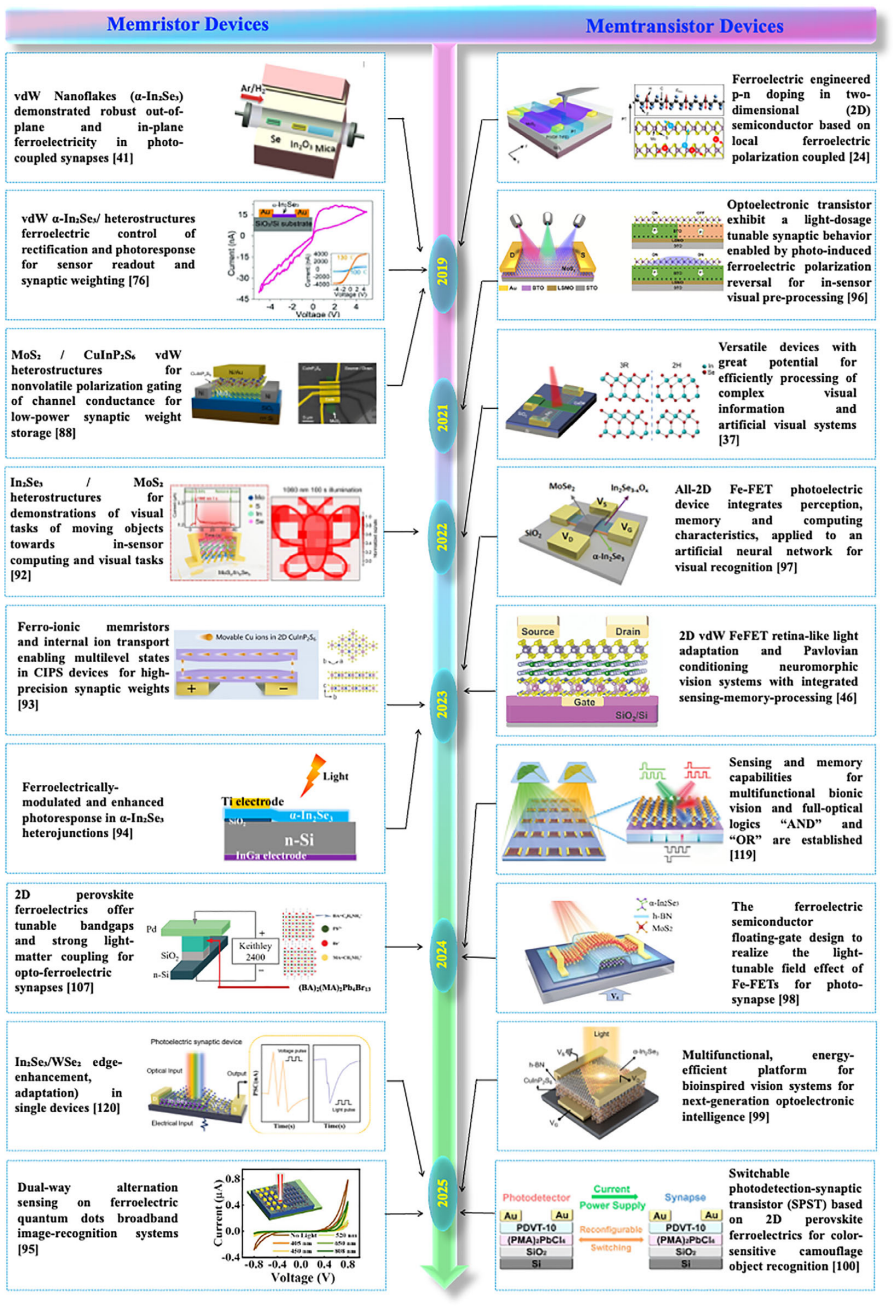
A timeline of the development of 2D ferroelectric materials‐based optoelectronic memristor and memtransistor devices for vision sensor applications. Reproduced with permission under the terms of the Creative Commons CC BY [24]. 2026, Springer Nature. Reproduced with permission [37]. 2026, John Wiley and Sons. Reproduced with permission [41]. 2026, American Chemical Society. Reproduced with permission under the terms of the Creative Commons CC BY [46]. 2026, John Wiley and Sons. Reproduced with permission [74]. 2026, John Wiley and Sons. Reproduced with permission [87]. 2026, American Chemical Society. Reproduced with permission [91]. 2026, American Chemical Society. Reproduced with permission [92]. 2026, Elsevier. Reproduced licensed under CC‐BY‐NC‐ND 4.0 [93]. 2026, American Chemical Society. Reproduced with permission [94]. 2026, John Wiley and Sons. Reproduced with permission [95]. 2026, Elsevier. Reproduced with permission [96]. 2026, Willey. Reproduced with permission [97]. 2026, AIP Publishing. Reproduced with permission [98]. 2026, American Chemical Society. Reproduced with permission [99]. 2026, Elsevier. Reproduced with permission Creative Commons CC BY [100]. 2026, John Wiley and Sons. Reproduced with permission [101]. 2026, John Wiley and Sons. Reproduced with permission [102 2026, Elsevier.

However, continued advances in material synthesis, encapsulation, and heterostructure engineering are expected to deliver fully in‐sensor neuromorphic imagers capable of performing complex visual tasks with ultra‐low power consumption. In the broader context of neuromorphic hardware, a 2D ferroelectric bionic vision sensor bridges optoelectronic transduction and cognitive processing, offering a tangible step toward human‐like visual intelligence implemented in solid‐state form. A timeline review for the development of 2D ferroelectric materials‐based optoelectronic vision sensors, including memristors and memtransistors, is depicted in Figure 5.

## 4 2D Ferroelectrics‐Based Memristive Devices for Retinomorphic Hardware

Memristors with two terminal electrodes have excellent potential as a synaptic core for hardware implementation in neuromorphic systems to integrate with CMOS technology due to their low variation, reliable analog switching, and conductance modulations [103]. Recently, optoelectronic memristors exhibiting resistive switching depending on the conductive filaments (CF) have been developed. Joule heating is a severe issue for the CF stability [104, 105, 106]. The ferroelectric materials have confirmed their performance for optoelectronic and information storage for their neuromorphic applications [107]. 2D ferroelectric semiconductor materials with vdWH offer unique properties and potential neuromorphic in‐sensor applications [108, 109].

**FIGURE 6:**
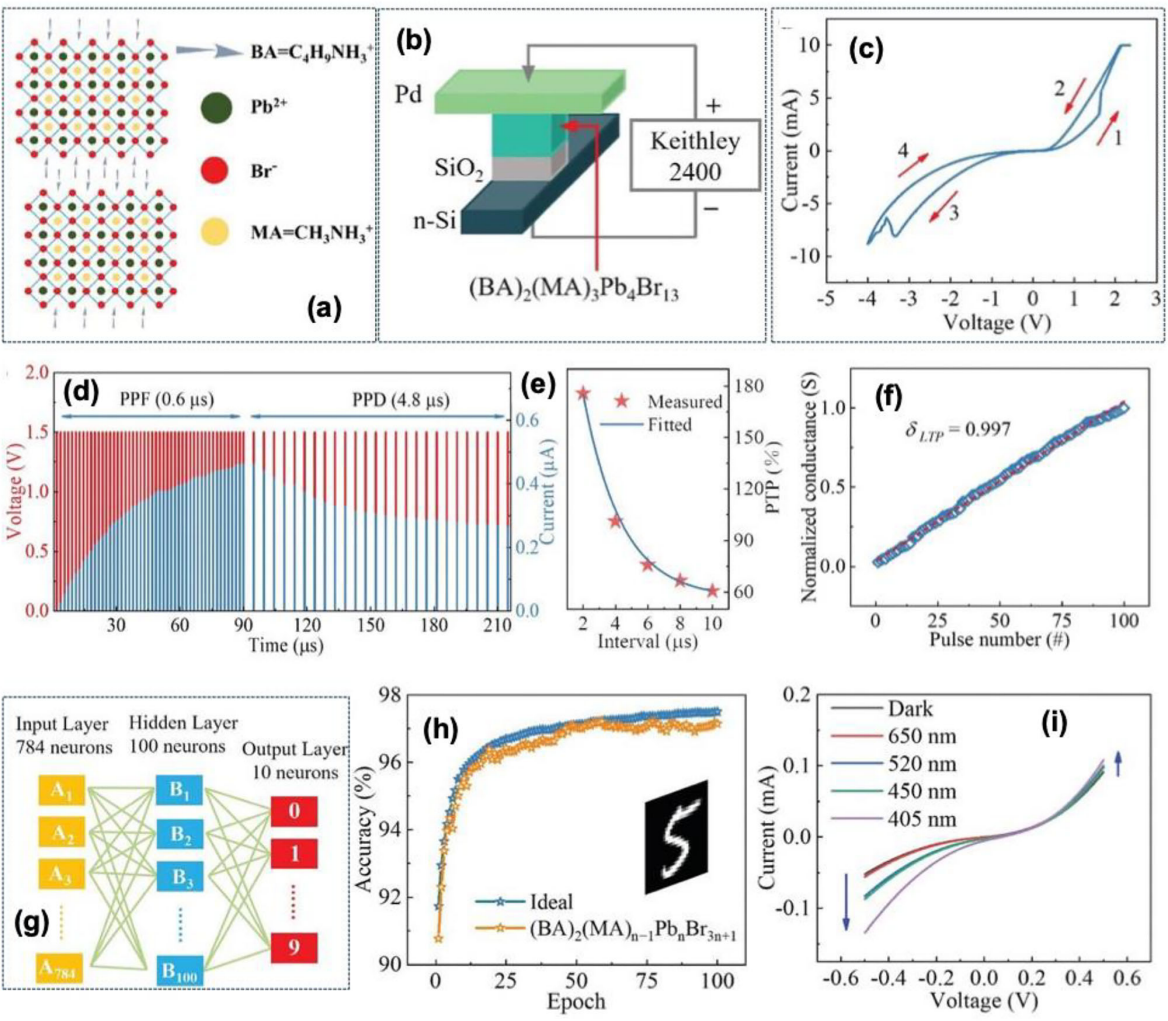
(a) 2D (BA)_2_(MA)_3_Pb_4_Br_13_ perovskite crystal structure, (b) Schematic diagram of the Pd/(BA)_2_(MA)_3_Pb_4_ Br_13_/SiO_2_/Si structure along with electrical measurement scheme, (c) bipolar I‐V characteristics of the fabricated device, (d) PPF (1.5 V, 0.6 µs) and PPD (1.5 V, 4.8 µs), (e) PTP test results and fitted curves, (f) LTP behavior of the devices, (g) designed artificial neural network consisting of 784 input and 10 output neuron along with 100 hidden layer, (h) recognition accuracy after 100 training sessions, including both ideal and actual situations, and (i) I‐V characteristic curves of devices under the light with different wavelengths (650/520/450/405 nm). Reproduced with permission under Creative Commons CC BY [100]. 2026, John Wiley and Sons.

Wang et al. [100] reported an optoelectronic memristor built on a 2D ferroelectric Ruddlesden‐Popper (RP) perovskite structure (BA)_2_(MA)_3_Pb_4_Cl_13_ (BA: C_2_H_9_NH_3_^+^; MA: CH_3_NH_3_^+^), configured in a Pd/(BA)_2_(MA)_3_ Pb_4_Cl_13_/SiO_2_/Si device architecture shown in Figure 6a,b. The device conductance can be coordinately modulated by both electrical pulses and optical stimuli, enabling an in‐sensor and in‐memory computing. The device concept directly targets a key limitation of conventional vision system, separated sensing‐memory‐computing, leading to high power and latency. The SiO_2_ interlayer of ∼ 3.4 nm was used to establish a controlled barrier height and minimize power consumption. Structural and compositional analyses such as X‐ray photoelectron spectroscopy (XPS), ultraviolet photoelectron spectroscopy (UPS), and PFM confirmed high crystallinity and robust ferroelectric behavior, with polarization reversal angles approaching 150° and coercive voltages of +1.0 V/−2.8 V. The residual polarization exhibited negligible decay after 10^9^ switching cycles, indicating excellent endurance. This 2D RP ferroelectric configuration provided a strong coupling between ferroelectric polarization and photoexcitation, which underpins the ability of the device to respond simultaneously to optical and electrical stimuli.

The device demonstrated bipolar resistive switching with stable high resistance state (HRS) and low resistance state (LRS) with more than 10^4^ s of retention under repeatable cycling. The current voltage characteristics exhibited SET and RESET transitions under bias sweeps of +2 V/−4 V, confirming non‐volatile memristive behavior referred to in Figure 6c. Electrical pulses in the microsecond regime produced dynamically tunable conductance modulation suitable for emulating multiple biological synaptic processes.

Under the application of successive voltage pulses (Amp: 1.5 V, pulse width: 1.2 µs, and interval: 0.6 µs), the device exhibited paired‐pulse facilitation (PPF) with gradually increasing current response. Extending the interval of 8.4 µs converted to the paired‐pulse depression (PPD), mimicking the neural transition from short‐term memory reinforcement to forgetting depicted in Figure 6d. Post‐tetanic potentiation (PTP) tests using 10 pulses (2 V, 1 µs) produced a PTP ratio of ≈ 100%, with relaxation time constants τ_1_ = 1.2 µs and τ_2_ = 2.0 µs, indicative of rapid yet stable synaptic reinforcement as shown in Figure 6e.

For long‐term plasticity, consecutive 100 pulses (3 V, 200 ns PW, 200–596 ns interval) yielded long‐term potentiation (LTP) with a nearly ideal linearity coefficient of ∼ 0.997, while reverse‐polarity pulses (−3 V, 1300 ns PW, 3300–330 ns interval) induces long‐term depression (LTD) with linearity of ∼ 0.770 in Figure 6f. This symmetric bidirectional conductance change is attributed to the non‐volatile ferroelectric polarization switching and stable mixed domain states in the 2D RP perovskite layer rather than conventional filamentary conduction. The device also reproduces a *learning–forgetting–learning* behavior consistent with the Ebbinghaus forgetting curve, after initial learning with 5 V stimuli, learning required significantly fewer pulses to restore the same conductance state, demonstrating efficient memory retention. The experimentally obtained LTP data were used as the weight matrices in a 2‐layer feed‐forward neural network trained on the MNIST handwritten digit dataset (784 × 100 × 10 neurons) to verify the neuromorphic simulation. The network achieved a recognition accuracy of 97.15% after 100 training epochs, underscoring the high linearity and analog weight tunability of the ferroelectric memristor Figure 6g,h.

**FIGURE 7:**
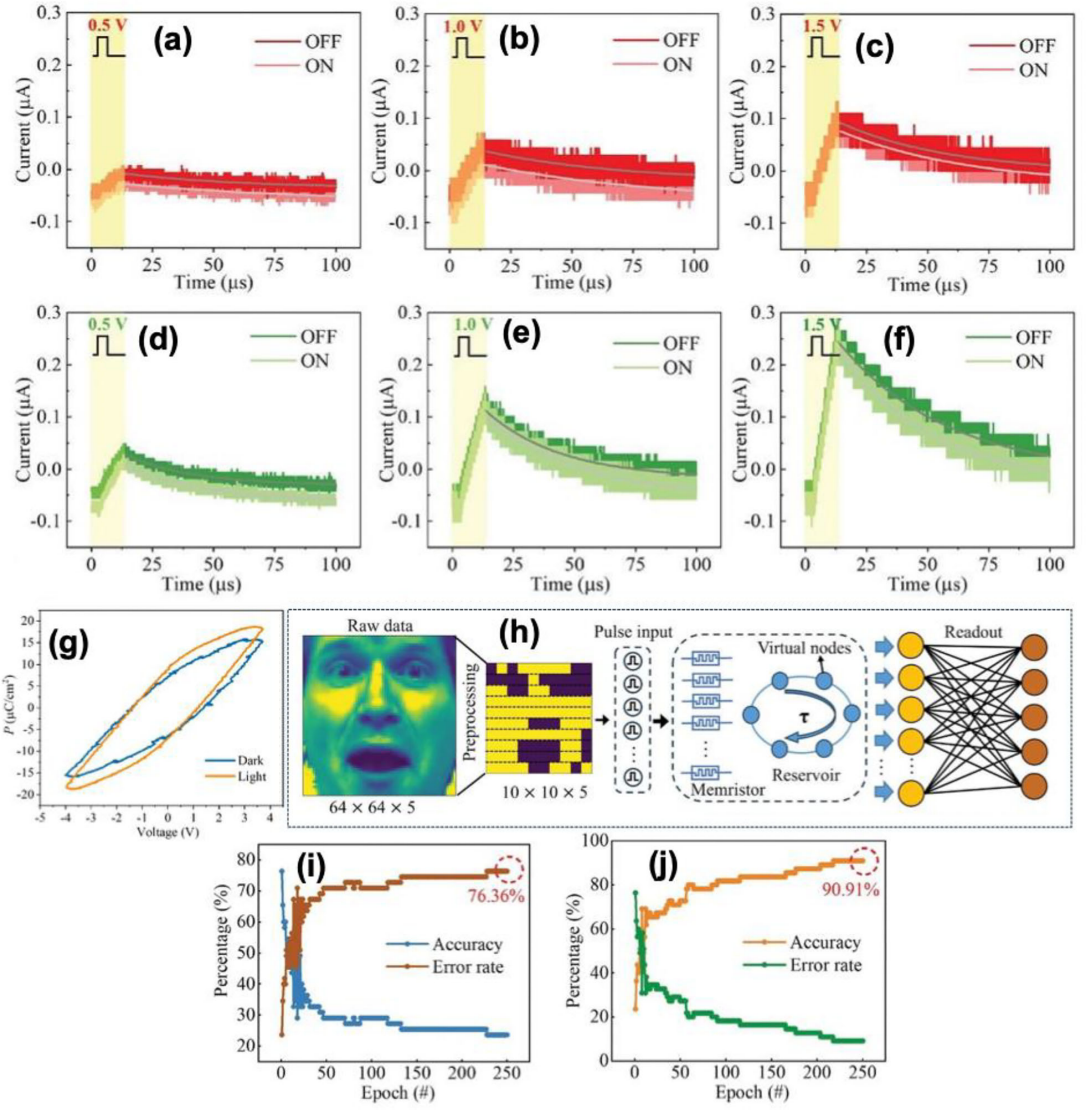
Current responses under different single electrical pulse and wavelength of 650 nm (a) 0.5 v, (b) 1.0 V, (c) 1.5 V, current responses under different single electrical pulse and wavelength of 520 nm (d) 0.5 V, (e) 1.0 V, (f) 1.5 V, (g) schematic of a neural network reservoir computation for image recognition, (h) Polarization‐electric‐field (P‐E) loops of 2D RP (BA)_2_(MA)_3_Pb4Br_13_ perovskite under the dark and illumination, (i) recognition accuracy and error rates of reservoir computing networks under electrical signals for scaled images, recognition accuracy and error rates of reservoir computing networks under electrical and optical signals for scaled images. Reproduced with permission under Creative Commons CC BY [100]. 2026, John Wiley and Sons.

The device I–V characteristics showed the wavelength‐dependent enhancement with stronger current in the negative bias region and an overall increase as the wavelength decreased, or illumination power increased under the influence of monochromatic light of 405 nm (blue), 520 nm (green), and 650 nm (red) shown in Figure 6i. Specifically at 650 nm illumination with power densities of 27.7, 138.9, and 277.8 mW cm^−2^, the current increased proportionally with intensity, revealing stable and reversible photoresponse behavior. With the application of 30 electrical pulses (0.5–1.5 V, 2 µs PW, 2 µs interval) under dark and illuminated conditions, the conductance increased markedly in the presence of light, demonstrating optically assisted potentiation as shown in Figure 7a–f. Excitatory postsynaptic current (EPSC) values increased by nearly twofold compared with dark conditions, and the relaxation time constant τ extracted from exponential decay fits of current responses became larger under illumination. So, at the higher wavelength, the device exhibited slower forgetting, whereas fast forgetting at the lower wavelength. Polarization–Electric field (P–E) loop measurements confirmed that the illumination enhanced the optical polarization. The polarization difference between light and dark states was ∼ 2.9 µC cm^−2^ (forward) and ∼2.7 µC cm^−2^ (reverse), depicted in Figure 7g. This coupling of ferroelectric polarization and photoexcitation underpins the optical‐electrical synergy in conductance modulation.

In neuromorphic‐vision demonstrations, the memristor served as a reservoir‐computing element. For face‐image recognition, a reservoir network using electrical inputs alone achieved 76.36% accuracy after 250 training iterations. Incorporating combined photo‐electrical inputs improves the accuracy to 90.91%, with a 14.55% enhancement, confirming the advantage of hybrid optical‐electrical stimulation for spatiotemporal feature encoding as shown in Figure 7h–j. The concept of optical‐electrical dual modulation is a strong step forward to event‐driven artificial vision where optical input can directly tune synaptic weights.

Despite its promising multifunctionality, the device operation was demonstrated only at RT. Testing under extended optical cycling or environmental stress should be done to show its potential in comprehensive operations. Moreover, the switching voltages and PW in the µs range are higher than those in state‐of‐the‐art low‐power oxide or 2D semiconductor memristors. Further study should be focused on lead‐free 2D ferroelectric systems, improved encapsulation, and lower operational voltage to enable integration into high‐density, energy‐efficient neuromorphic vision arrays.

**FIGURE 8:**
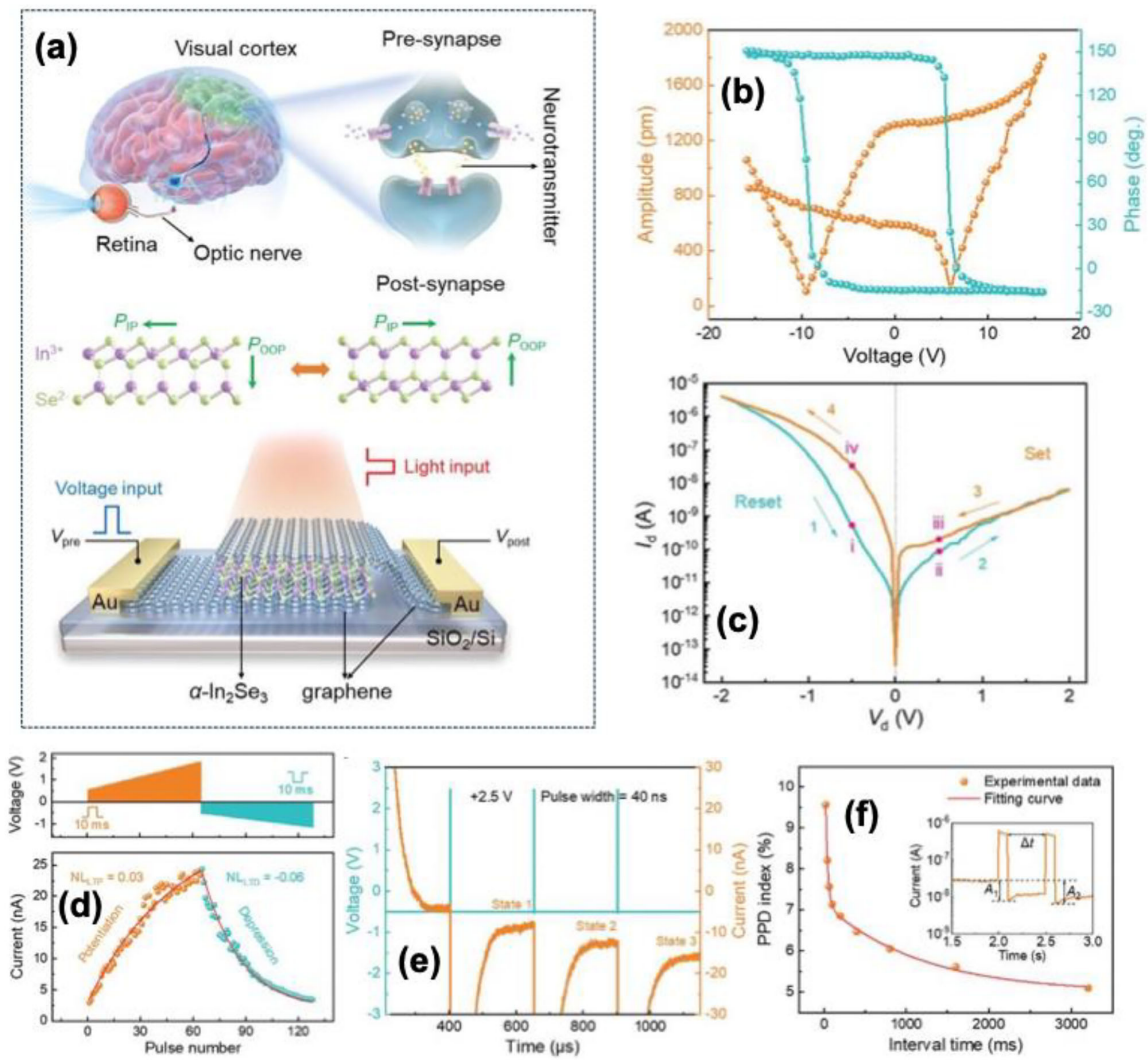
(a) Top: schematic of the human visual perception and processing system and enlarged synaptic structure. Center: crystal structures of 𝛼‐In_2_Se_3_ highlight the dipole locking effect in both IP and OOP orientations. Bottom: corresponding schematic of graphene/𝛼‐In_2_Se_3_/graphene crossbar‐structured optoelectronic synapse, designed for electrical and optical regulation. (b) Hysteretic behavior of the OOP PFM amplitude and phase was observed by applying bias on the tip, demonstrating the ferroelectric polarization switching process. (c) I_d_‐V_d_ characteristics of the 𝛼‐In_2_Se_3_ device displaying a hysteresis loop due to OOP ferroelectric polarization switching. States i–iv show currents at different polarization directions at ±0.5 V, (d) With several 40 ns (+2.5 V) write spikes, the device's conduction states evolve, highlighting ultrafast programmability, (e) LTP using 64 pulses (ramping from +0.56 to +1.82 V, 10 ms) and LTD with 64 pulses (ramping from −0.52 to −1.15 V, 10 ms) under a −0.5 V bias for state reading. The red line represents the result obtained from fitting using the nonlinearity formula, and (f) PPD index plotted against pulse interval, calculated as (A_2_‐A_1_)/A_1_ × 100%. The red curve illustrates fitting results from the double exponential decay function. Inset: PPD effect schematic with definitions of A_1_, A_2_, and time interval Δt.Reproduced with permission [110]. 2026, John Wiley and Sons.

In the preceding, Zeng et al. [110] reported a multifunctional ferroelectric semiconductor synapse that simultaneously senses, memory, and provides computing capabilities within a graphene/*α*‐In_2_Se_3_/graphene crossbar configuration as shown in Figure 8a. This introduces a multisensory ferroelectric semiconductor synapse capable of integrating multiple stimulus modalities, not only electrical, particularly designed for neuromorphic computing tasks. The device demonstrates that the ferroelectric semiconductors can support synaptic plasticity behaviors needed for neuromorphic computing.

**FIGURE 9:**
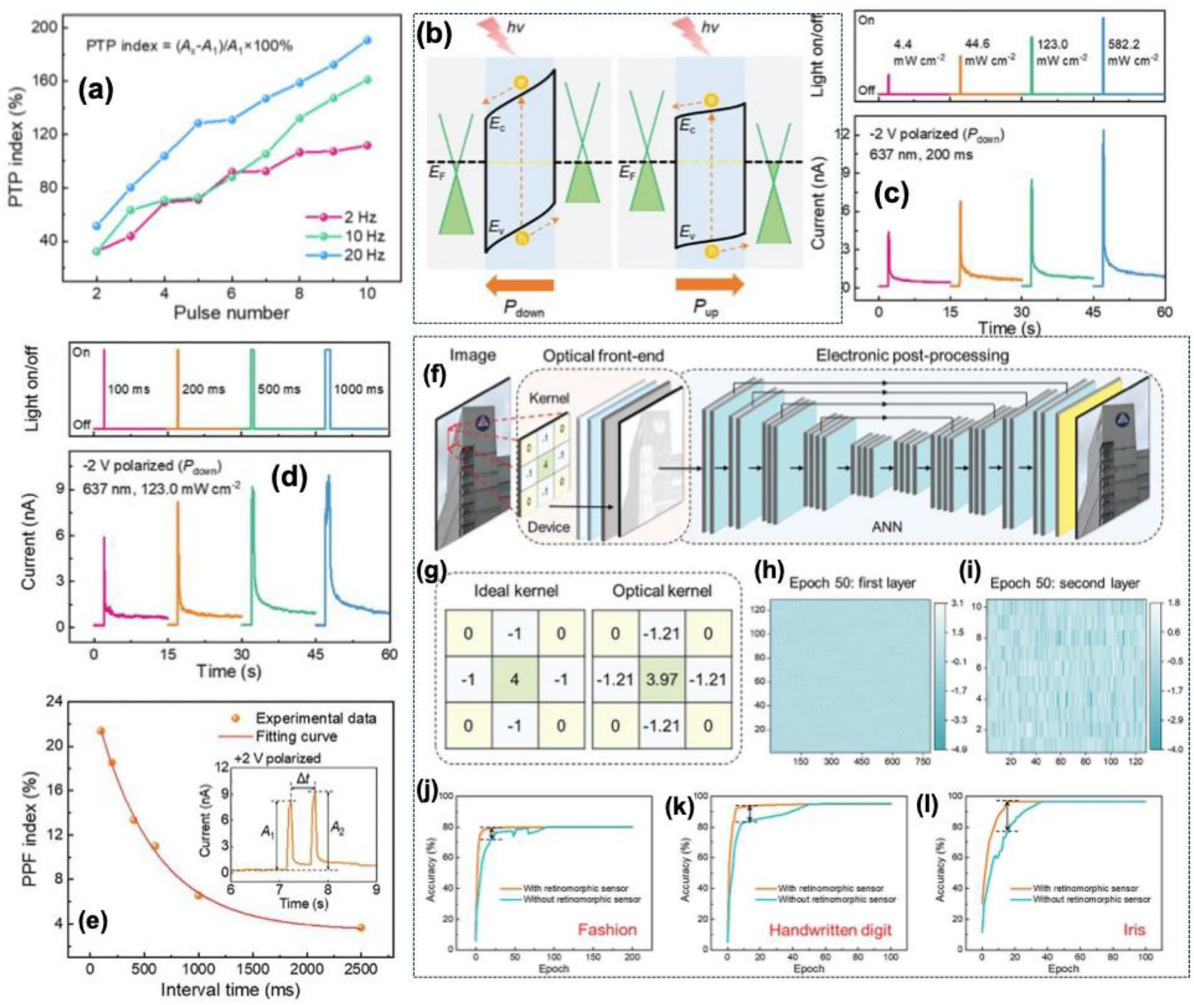
(a) PTP characteristics at varying frequencies, (b) Energy band diagrams for the 𝛼‐In_2_Se_3_ device under varied polarization states during light illumination, highlighting the ferroelectric polarization's impact on the generation and movement of photogenerated carriers, (c) PSC profiles in response to optical pulses with varied amplitudes (4.4, 44.6, 123.0, and 582.2 mW cm^−2^, 200 ms) on the device in its P_down_ state, utilizing a 0.1 V bias for state reading, (d) PSC profiles in response to optical pulses with diverse widths (100, 200, 500, and 1000 ms, 123.0 mW cm^−2^) on the device in its P_down_ state, using a 0.1 V bias for state reading, (e) PPF index versus pulse interval, given by (A_2_ − A_1_)/A_1_ × 100%. The red curve denotes the fitting using a double exponential decay function. Inset: schematic of light‐induced PPF effect, and definitions of A_1_, A_2_, and time interval Δt, (f) Extended ANN model featuring an optical front‐end layer with a 3 × 3 convolution kernel that utilizes the device's non‐volatile photoresponsivity and an electronic post‐processing domain with three layers of fully‐connected neural networks that utilizes the device's non‐volatile conductance, (g) Comparison between actual optical kernel and ideal convolution kernel values. (h,i) Final weights of the first and second layers after 50 training epochs, and (j–l) Comparative analysis of pattern recognition accuracy for Fashion, Handwritten digit, and Iris datasets, with and without the retinomorphic sensor. Reproduced with permission [110]. 2026, John Wiley and Sons.

The *α*‐In_2_Se_3_ crystal features a Se–In–Se–In–Se quintuple layer sequence, exhibiting a dipole‐locking effect and robust out‐of‐plane ferroelectricity depicted in Figure 8a. Piezoresponse force microscopy (PFM) confirmed a 180° phase reversal with a butterfly‐shaped amplitude loop, verifying switchable polarization as shown in Figure 8b. Work function of 4.11 eV (*α*‐In_2_Se_3_) and 4.81 eV (graphene), forming polarization‐tunable Schottky junction at the interface. Under the dark condition, the device exhibited a rectifying hysteresis with an ON/OFF ratio of ∼10^2^ shown in Figure 8c. The device supports 12 distinct conductance states, demonstrating analog multilevel storage capability. Electrical programming and erase using ±2.5 V, 40 ns PW achieved ultrafast switching, confirming rapid ferroelectric domain reversal as shown in Figure 7d. Linear LTP and LTD were demonstrated with a nonlinearity coefficient (NL) of +0.03 and −0.06, respectively, indicating symmetric and linear weight update suitable for neuromorphic learning shown in Figure 8e. These behaviors arise from gradual ferroelectric domain switching that modulates the Schottky barrier height and hence the channel conductance. Temporal learning functionalities were further verified through PPD and spike‐rate‐dependent plasticity (SRDP). When two successive electrical spikes were applied, the second EPSC decreased depending on the inter‐pulse interval (Δt). The PPD index reached approximately 80% for short intervals (Δt = 10 ms) and followed a double‐exponential decay with relaxation constants τ_1_ ≈ 80 ms and τ_1_ ≈ 420 ms, shown in Figure 8f. Similarly, the device exhibited frequency‐dependent facilitation analogous to PTP with increasing potentiation at higher spike rates (2 to 20 Hz) shown in Figure 9a. Energy band diagrams for the 𝛼‐In_2_Se_3_ device under varied polarization states during light illumination, highlighting the ferroelectric polarization's impact on the generation and movement of photogenerated carriers shown in Figure 9b. The intrinsic optoelectronic properties were verified under a 637 nm laser and 0.1 V read bias; the device displayed significant modulation of photocurrent and photoresponsivity as a function of ferroelectric polarization. The responsivity α = 0.37 (upward polarization) and α = 0.28 (downward polarization), indicating a trap‐mediated photogating effect. The device exhibited broadband spectral sensitivity from 340 to 940 nm, with a maximum responsivity up to ≈350 A W^−1^ under 520 nm illumination and an ON/OFF ratio ∼10^3^. The photoresponsivity could be linearly tuned through ferroelectric domain reorientation via electrical pulses (−0.80 C to −1.8 V, 100 ms PW), demonstrating optically controlled LTP of synaptic weights. The devices with different polarization directions display EPSC characteristics akin to electronic synapses. For a constant pulse width of 200 ms and varying amplitudes (4.4, 44.6, 123.0, and 582.2 mW cm^−2^), the peak photocurrent rises with light intensity shown in Figure 9c.

The variation of optical PW from 100 to 1000 ms further confirmed the modulation of carrier trapping and release dynamics as shown in Figure 9d. Optical PPF behavior was observed when two consecutive light pulses were applied; the second EPSC peak exceeded the first for a short inter‐pulse interval. The PPF index reached ∼125% at *Δt* = 10 ms and decayed exponentially with increasing *Δt*, exhibiting two relaxation times of τ_1_ ≈ 95 ms and τ_1_ ≈ 510 ms as shown in Figure 9e. This strong temporal correlation under light stimulation demonstrated the feasibility of *α*‐In_2_Se_3_‐based optoelectronic synapses for light‐modulated plasticity and visual sensory learning.

The neuromorphic vision performances were evaluated using a hybrid simulation framework coupling optical front‐end convolution and electronic post‐synaptic processing using experimentally calibrated device parameters. The front‐end 3 × 3 convolution kernel utilized non‐volatile photoresponsivity values ranging from −1 to 4 A W^−1^ for edge enhancement of 28 × 28‐pixel input images, while the back‐end fully connected artificial neural network (ANN) employed a 784 input nodes × 128 hidden nodes × 10 output nodes architecture using the conductance states as trainable synaptic weights depicted in Figure 9f–i. This hybrid network effectively replicated the biological visual system, performing image processing (retina‐like) and classification (cortex‐like) concurrently. Recognition accuracy reached 80% for Fashion‐MNIST, 95% for handwritten digits, and 97% for IRIS recognition datasets, as shown in Figure 9j–l. The inclusion of the optical preprocessing layer significantly accelerated network convergence and improved final accuracy compared to a conventional ANN architecture without sensory integration.

The device performance goes beyond “single‐mode synapses” by aiming for multisensory fusion, which is a major future direction for neuromorphic hardware that is closer to biological perception.

Despite significant advances, the study primarily focuses on device‐level demonstration under controlled experimental conditions and leaving the challenges in large‐scale integration, uniformity, and crossbar variability. The device can suffer polarization fatigue during long‐term operation, which can impact the stability of synaptic weight modulation. The optical sensing capability is limited to specific wavelength ranges and intensity levels that restrict its application scope in the multisensory perception systems and thermal management within the heterostructure could become critical during continuous operation. The proof‐of‐concept results show promising neuromorphic and sensory functionalities. However, system‐level integration, array scalability, and CMOS compatibility are essential to translate this approach into functional in‐sensor or neuromorphic vision hardware platforms.

**FIGURE 10:**
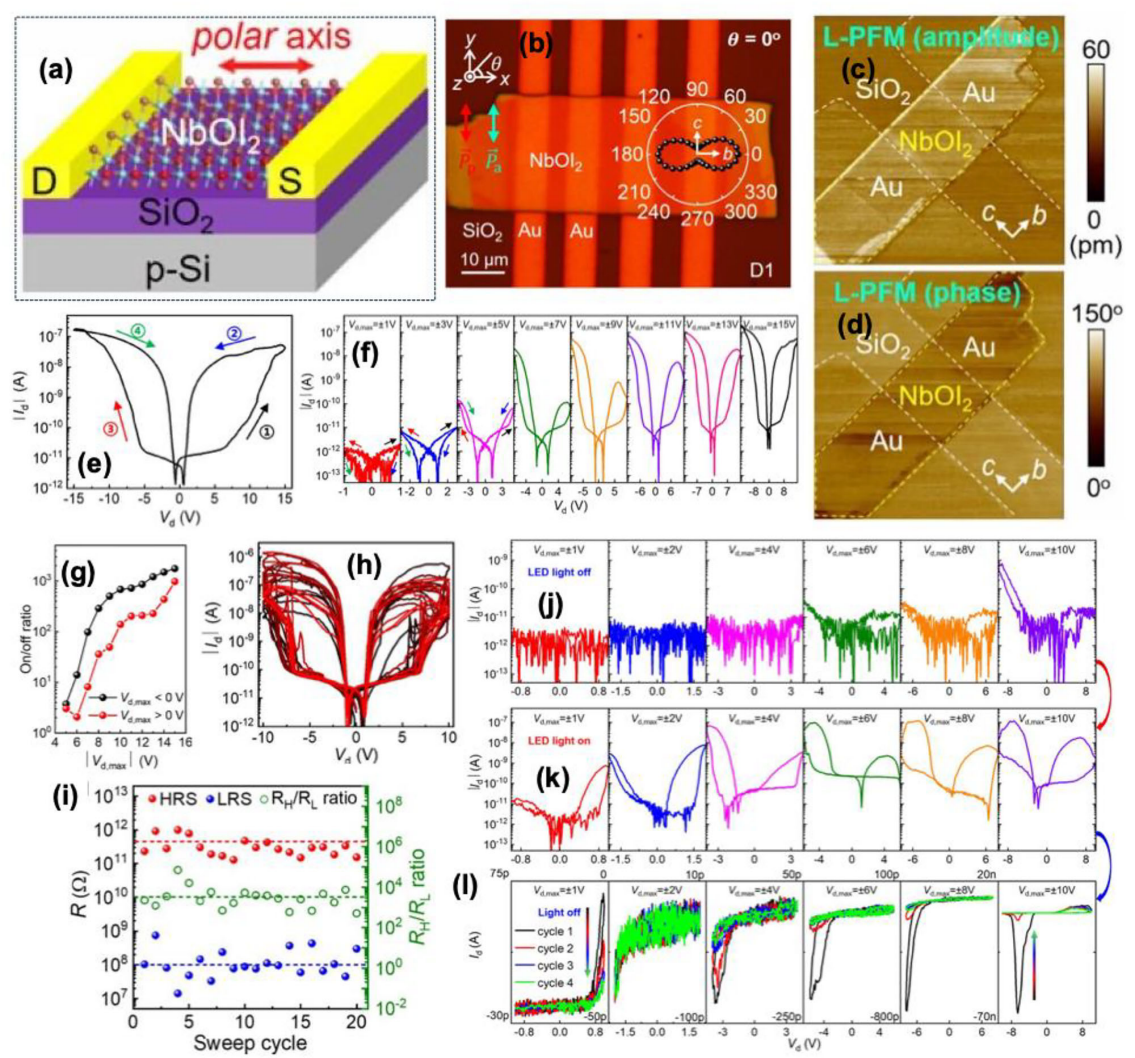
(a) Schematic of NbOI_2_ FET device using SiO_2_ as the bottom gate, where the polarization direction (*b*‐axis) of NbOI_2_ is perpendicular to two Au electrodes, (b) Optical image of a multilayer NbOI_2_ device (∼45 nm). Insets: (left) the laboratory coordinate system and the incident light (analyzer) polarization; (right) polar plot of parallel polarized light intensity as a function of the sample rotation angle, (c) phase images of a planar NbOI_2_ device. Insets: The in‐plane crystal orientation of 2D NbOI_2_, (d) Schematic band diagram of the ferroelectric switching mechanism, (e) Output (*I*_d–_ *V*_d_) characteristic (*V*_d,max_ = ±15 V) of the device in panel, (f) *I*_d–_ *V*_d_ curves of the NbOI_2_ device taken at different *V*_d,max_, (g) Relationship between the *I*_on_/*I*_off_ ratio and *V*_d,max_, (h) *I*_d–_ *V*_d_ curves of the NbOI_2_ device during 20 sweep cycles, (i) Statistical distribution of (left) resistances in the HRS (*R*_H_, red dots) and LRS (*R*_L_, blue dots) and (right) *R*_H_/*R*_L_ ratio (green dots) during the 20 cycle process in panel, (j,k) *I*_d–_ *V*_d_ curves of the device in panel (j) taken in the dark (k) and under 610 nm LED light exposure at different *V*_d,max_. (l) *I*_d–_ *V*_d_ curves of the device in panel taken when the LED light turned off again, with a cycling period of ∼180 s. Reproduced with permission [111]. 2026, American Chemical Society.

Hao et al. [111] further reported an in‐plane 2D ferroelectric niobium oxide diiodide (NbOI_2_) nanosheet‐based memristor, with both intrinsic electrical switching and visible‐light‐enhanced conductance modulation. The device employed mechanically exfoliated NbOI_2_ flakes of 50 to 80 nm thickness and transferred on the Al_2_O_3_/Si substrate, illustrated in Figure 10a,b. The remnant polarization amplitude reached ∼7.3 µC cm^−2^, with coercive voltages near ±2.1 V, confirming robust ferroelectricity in the lateral configuration as shown in Figure 10c,d. The device's lateral geometry reduced depolarization fields and facilitated direct light exposure. The demonstrates memristive behavior in in‐plane ferroelectric NbOI_2_, and importantly exhibited LED‐light enhanced memristor performance, linking the ferroelectric switching and photoexcitation. The LED‐enhances operation provides a pathway toward optoelectronic synapses with tunable plasticity.

The device exhibited analog resistive switching driven by ferroelectric polarization reversal, without reliance on electrochemical filament formation, depicted in Figure 10e–h. The stable memory window of approximately 10^2^–10^3^ with the retention of 10^4^ s and 10^3^ electrical cycles is shown in Figure 10i. The conduction mechanism analysis revealed a transition from trap‐limited space charge‐limited current (SCLC) in the HRS to a polarization‐enhanced conduction regime in the LRS. Short‐term plasticity behavior was successfully emulated by PPF under two sequential voltage pulses (1.5 V, 100 ms PW), the second EPSC increased by ≈ 160% relative to its first, and the PPF index decayed exponentially with inter‐pulse interval of (Δt) with a time constant τ ≈ 55 ms, as shown in Figure 10j–l. LTP and LTD were achieved by sequentially applying 20 positive and 20 negative pulses (2 V, 50 ms PW), resulting in gradually increasing and decreasing conductance states, respectively. The linearity coefficients for potentiation and depression were 0.987 and 0.975, demonstrating near‐ideal analog weight update capability. In neuromorphic simulations using experimentally calibrated conductance profiles, the device achieved 95.2% recognition accuracy on the MNIST dataset, underscoring its reproducible and symmetric conductance modulation characteristics.

The device exhibited strong photoresponsive modulation under white LED illumination (intensity range 0–120 mW cm^−2^) as shown in Figure 10j–l. The photocurrent increased monotonically with light intensity, producing up to three times enhancement in ON‐state conductance compared to dark conditions. Under illumination, the device's I–V hysteresis expanded symmetrically, and switching voltage decreased to +1.5 V/−1.8 V, evident in photo‐induced barrier lowering due to photocarrier screening of the polarization field shown in Figure 10k. EPSC measurements under 2 V pulse revealed that the light‐assisted current reached ≈220 nA, nearly double the dark EPSC of ≈115 nA, with an extended relaxation constant (τ_dark_ ≈45 ms to τ_light_ ≈96 ms). The device achieved stable multilevel conductance states under periodic light and electrical co‐stimulation, suggesting potential for event‐driven sensing‐memory integration in neuromorphic vision applications. The device significantly incorporated in‐plane ferroelectricity that allows the planer device designs with complex integration schemes. The device performance based on the polarization‐controlled conduction without relying on vertical stacks.

Despite its impressive optoelectronic synergy, the switching voltages (∼2 V) remain moderate, but still higher than sub‐volt operation achieved in optimized 2D or HfO_2_‐based ferroelectric devices. The endurance (∼10^3^ cycles) and long‐term ambient stability of the device are constrained by surface oxidation and interlayer degradation. Additionally, the spectral response relies on broadband illumination, which is less selective than wavelength‐tuned photonic devices. Future study would be done on wafer‐scale growth of NbIO_2_ thin films, encapsulation strategies to enhance environmental robustness, and vertical device architectures to improve scalability. The possible integration of NbIO_2_ with optical waveguides or CMOS‐compatible photonic circuits could enable a high‐speed, low‐power neuromorphic visual processor that leverages its intrinsic photoelectric‐photoconductive coupling.

**FIGURE 11:**
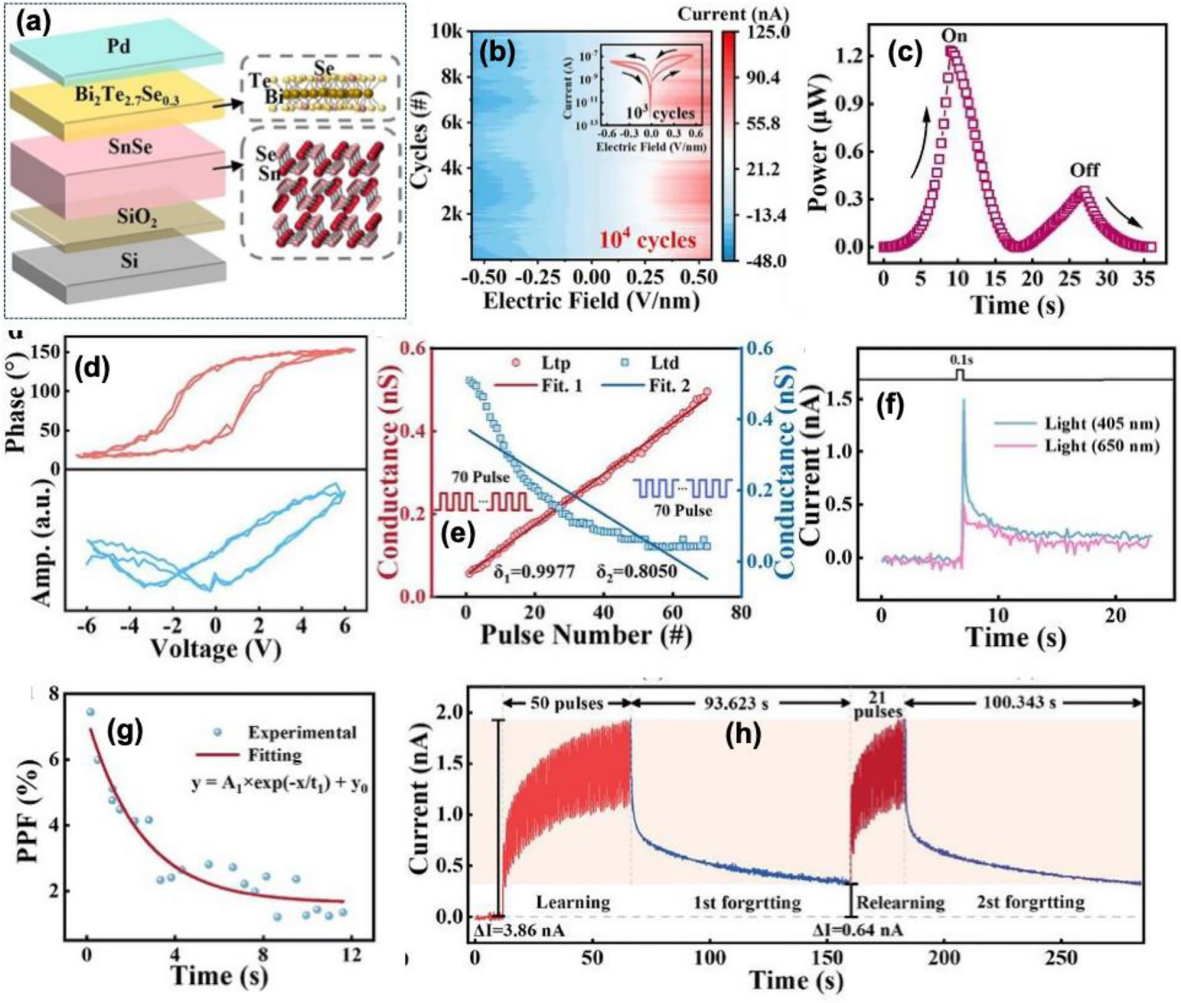
(a) Architecture and composition for Bi_2_Te_2.7_Se_0.3_/SnSe hetero‐memristors, (b) Highly stable logarithmic I–V contour diagram of the hetero‐memristor (10^4^ cycles), the inset shows I–V hysteresis curves (10^3^ cycles), (c) Power of the device during “turn‐on” and “turn‐off.” (d) Out‐of‐plane ferroelectric of 2D SnSe films including the PFM phase and amplitude curves, (e) witching behaviors from the long‐term potentiation to long‐term depression of electrical synapse, (f) transient current response of the hetero‐memristor under different optical signals (405 nm/650 nm), (g) Variations of PPF index with optical pulse intervals, and (h) Learning‐forgotten‐relearning behaviors (50 pulses → 93.62 s → 21 pulses → 100.34 s) of the analog human brain. Reproduced with permission [112]. 2026, John Wiley and Sons.

Wang et al. [112] reported a high efficiency and stable heterostructure memristor based on 2D topological insulator bismuth tellurium selenide (Bi_2_Te_2.7_Se_0.3_) and 2D ferroelectric SnSe as shown in Figure 11a. The vertically stacked Pd/Bi_2_Te_2.7_Se_0.3_/SnSe/SiO_2_/Si exhibits ferroelectric polarization‐driven charge modulation in SnSe synergistically interacts with the Dirac surface state of Bi_2_Te_2.7_Se_0.3_ to tune charge transport properties. This work proposes a heterostructure memristor combining topological Bi_2_Te_3_ and ferroelectric SnSe, particularly designed for in situ bionic‐visual semi‐hardware systems. The device integrates the topological conduction advantages, that potentially provides a stable transport channels with ferroelectric switching for memory and synaptic behavior. The engineered band offset as discussed in Section 2.2, giving rise to effective resistive switching with continuous conductance tunability over 25 stable states, a highly reproducible I—V hysteresis cycle up to 10^4^ cycles and HRS/LRS retention exceeds 10^4^ s, including stable retention at elevated temperature (85°C) shown in Figure 11b. The switching power consumption is exceptionally low, averaging ∼0.25 µW in both SET and RESEST operations in Figure 11c. The SnSe layer (∼11.7 nm) offered robust out‐of‐plane ferroelectricity with a coercive voltage of ∼3 V and polarization phase shift of ∼140°, whereas the Bi_2_Te_2.7_Se_0.3_ layer (∼4.4 nm) provides a photoactive topological conduction channel shown in Figure 11d. Linearity in LTP reaches 0.9977, supporting accurate synaptic weight updates as shown in Figure 11e. The device successfully emulates various biological synaptic functionalities such as PPF, post‐tetanic potentiation (PTP), spike‐timing‐dependent plasticity (STDP), enabling reliable low‐powered neural computation.

A notable advancement is its optoelectronic synaptic function, where the heterostructure efficiency converts incident photons into conductance modulation. Short‐pulse optical stimuli (405 nm/650 nm) induce rapid EPSC responses shown in Figure 11f, while variation in PW, duration, frequency, and spacing allowed the device to emulate short‐term memory (STM) to long‐term memory (LTM) transitions. Double‐pulse optical stimulation achieves PPF and switching to suppression (PPD) by tuning inter‐pulse spacing or frequency, confirming controllable plasticity in purely light‐stimulated regimes shown in Figure 11g. The maximum photocurrent modulation (cI) reaches ∼2.8–3.86 nA, showing fatigue‐resistant *learning–forgetting–learning* behavior analogues to biological visual cognition as shown in Figure 11h.

**FIGURE 12:**
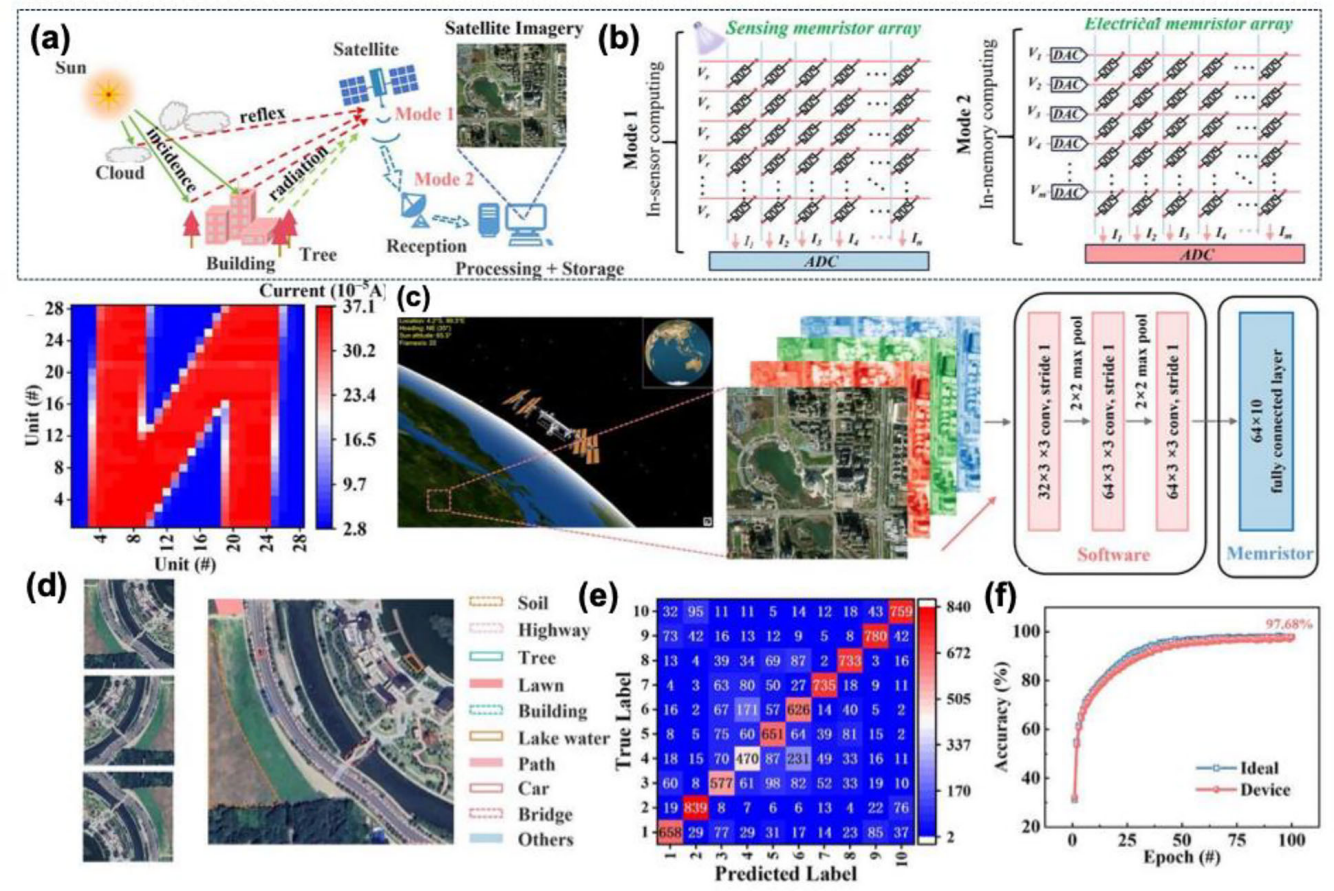
(a) Composition diagram of the satellite system, (b) Circuit diagrams composed of optical (Mode 1) and electrical (Mode 2) hetero‐memristor arrays, (c) Schematic diagram of the convolutional neural network (CNN), (d) Example image after treatment of features extracted from a single object during image recognition. The image on the left is an example of flipping a pre‐recognized image. The flipping angles are 0°, 90°, and 180°, respectively. (e) Confusion matrix for predicted label and true label of image recognition results. (f) Recognition accuracy after 100 iteration cycles for the semi‐hardware systems in both ideal (Blue) and practical (Pink) situations. Reproduced with permission [112]. 2026, John Wiley and Sons.

The composition diagram of the satellite system and the circuit diagrams composed of optical (Mode 1) and electrical (Mode 2) hetero‐memristor arrays is shown in Figure 12a,b. Leveraging these properties, the construction of a 28 × 28 hetero‐memristor perception arrays functioning as an in‐sensor and in‐sensor computing platform for satellite image recognition, as shown in Figure 12c. The system achieves an image classification accuracy of 97.68% after 100 training iterations through a convolutional neural network (CNN) co‐processing as shown in Figure 12d–f. This confirms the feasibility of utilizing hetero‐memristors in semi‐hardware machine vision, particularly where co‐localized sensing, memory, and computation are imperative for energy‐efficient edge intelligence. The device performance explicitly targets stable and efficient visual computing and overcoming a key weakness of many oxide and filamentary memristors.

Although the Bi_2_Te_2.7_Se_0.3_/SnSe hetero‐memristor marks a significant step toward integrated neuromorphic vision hardware, the device‐to‐device (D2D) variation within large area memristor arrays is not fully quantified, which could impact uniformity in multi‐level synaptic weight mapping. The operational voltage ∼ ±6 V is still relatively high including material synthesis via pulsed‐laser deposition and magnetron sputtering may face challenges in large‐scale uniformity and wafer‐level integration. Additionally, long‐term optical reliability under high‐frequency illumination and the response in broadband visible/IR environments require deeper investigation for real‐world machine‐vision challenges for CMOS BEOL‐compatible, array yield improvements.

Wang et al. [113] further demonstrated a multifunctional memristive device based on the bismuth (Bi)‐doped SnSe within a compact two‐terminal Au/Sn_1−x_Bi_x_Se/NSTO structure to establish stronger and more symmetric out‐of‐plane polarization, resulting in the balanced coercive voltage of ±2.4 V, which is crucial for linearly symmetric conductance modulation needed in weight update rules. The device demonstrates a 2D ferroelectric memristor that integrates all‐in‐one sensing‐memory‐computing, for compact neuromorphic in‐sensor computing. This exhibited a strong integrated system‐level demonstrations, rather a separate photodetector, memory, processor.

**FIGURE 13:**
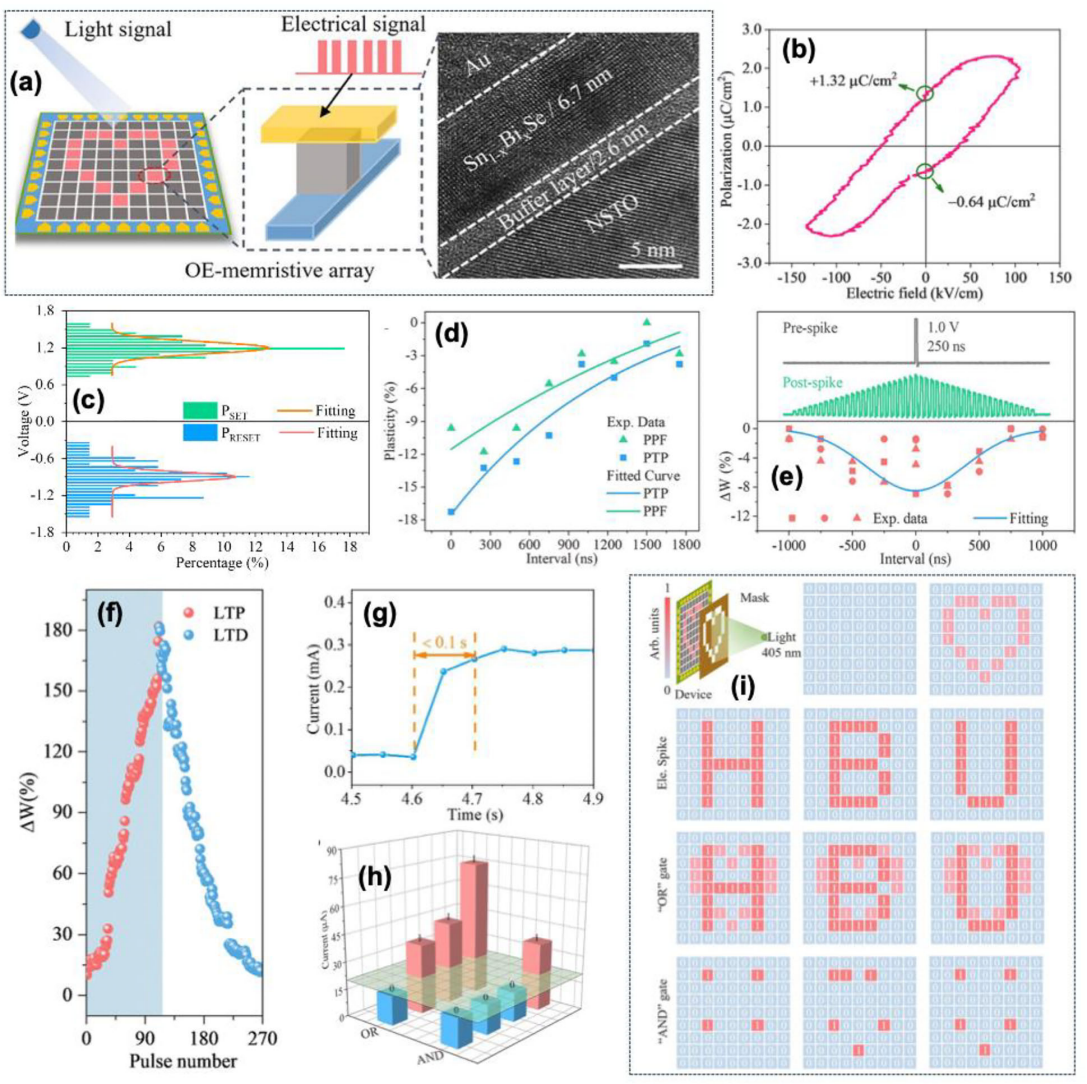
(a) The schematic diagram of the Au/Sn_1−x_Bi_x_Se/NSTO memristor array (left) and the illustration is the TEM image for the device cross‐section (right), (b) *P‐E* loop of fabricated device, (c) SET/RESET voltage distribution of multiple devices, (d) Short‐term plasticity of PPF and PTP synaptic functions, (e) Long‐term plasticity of the symmetrical STDP for synapse functions, (f) Realization for synapse characteristics including the optical LTP and electrical LTD, (g) Response speed of the Sn_1−x_Bi_x_Se device for the optical signals (405 nm, 30 mW/cm^2^), (h) Output current of “OR” and “AND” logic gates under photoelectric signals, (i) Output patterns of memristor array with 9 × 9 units after applying the input signals and computing. Schematic of the input pattern light. The initial states of the memristive array. The output image of the “heart” shape for light sensing and memory. The output images of “HBU” letters for electrical signal writing and memory. The “OR” and “AND” logic output images for computing and memory by integrating “heart” and “HBU”. Reproduced with permission [113]. 2026, Elsevier.

The deposited Sn_1−x_Bi_x_Se thin film exhibited a thickness of ∼6.7 nm, verifying the 2D nature and crystallinity suitable for ultra‐scaled neuromorphic architecture shown in Figure 13a,b. The memristive device showed the bipolar resistive switching with SET and RESET transition at +1.2 V and −0.9 V, while the switching is non‐filamentary and controlled by ferroelectric polarization‐induced band modulation with multi‐bit storage essential for analog neuromorphic hardware as shown in Figure 13c. The device demonstrates PPF of approximately 11.5% and PTP up to 17.3%, confirming the STP behavior responsible for learning speed and signal filtering in biological synapses shown in Figure 13d. The memristor also exhibits symmetric STDP, enabling bidirectional long‐term weight modification independent of spike order, which is desirable for stability in training as shown in Figure 13e. Highly linear and symmetric LTP/LTD characteristics are obtained with minimal state overlap due to the symmetric ferroelectric coercive field, signifying high‐precision learning capability for classification tasks in future neuromorphic networks depicted in Figure 13f. In addition to synaptic learning, neural signal integration and firing are successfully emulated within the same device. Both temporal integration and spatial summation generate threshold‐driven firing events, eliminating the need for external neuron circuits. This simultaneous realization of synapse‐to‐neuron fusion strengthens device‐level computational density.

The optoelectronic sensing for neuromorphic vision with a 405 nm light source, the device demonstrates a rapid photoresponse of approximately 0.1 s, followed by a stable nonvolatile photoconductance retention even after the optical stimulus is removed, as shown in Figure 13g. Additionally, the memristor supports 8 clearly distinguishable photocurrent states, enabling multilevel optical memory storage within a single device.

Real‐time optical image sensing, in situ storage, and logic computing (OR/AND) are experimentally demonstrated at the array level, and the corresponding current response is shown in Figure 13h. The performance was further validated in a 9 × 9 memristor array, where in‐sensor pattern acquisition and processing are achieved as shown in Figure 13i. 3‐bit octal demodulation of optical signals was achieved with different light intensities encoding ASCII‐mapped characters (‘LOVE’), supporting low‐latency edge intelligence without ADC/DAC interface. Bi doping is presented as a method to tune ferroelectric and memristive properties, leading the devices improved reliable neuromorphic functions. The device offers a potential direction to compact neuromorphic vision hardware, leveraging the synergy of ferroelectric polarization and photoexcited carrier trapping to unify perception, memory, and computing in one integrated nanoscale element.

However, the device has limitations, including a relatively small intrinsic polarization (∼2.42 µC cm^−2^), moderate optical response speed compared to high‐speed photonic synaptic devices, and the absence of high‐cycle endurance or long‐term retention studies at the array level for the integration with CMOS back‐end platforms.

## 5 2D Ferroelectrics‐Based Memtransistor Devices for Retinomorphic Hardware

In recent years, various optical sensing and synaptic devices have been combined to realize artificial visual systems based on two‐terminal architectures [114, 115]. In such systems, optical responses mainly originate from band‐to‐band excitation or the electron trapping process [116, 117]. Consequently, they typically exhibit single‐wavelength sensitivity, narrow spectral bandwidth, and complex device layouts [118]. To achieve filter‐free broadband color detection within a single unit, a new design approach is needed. Furthermore, employing a compact three‐terminal memtransistor configuration can simplify the structure while enabling gate‐controlled modulation of light‐induced effects for multifunctional processing within single device.

**FIGURE 14:**
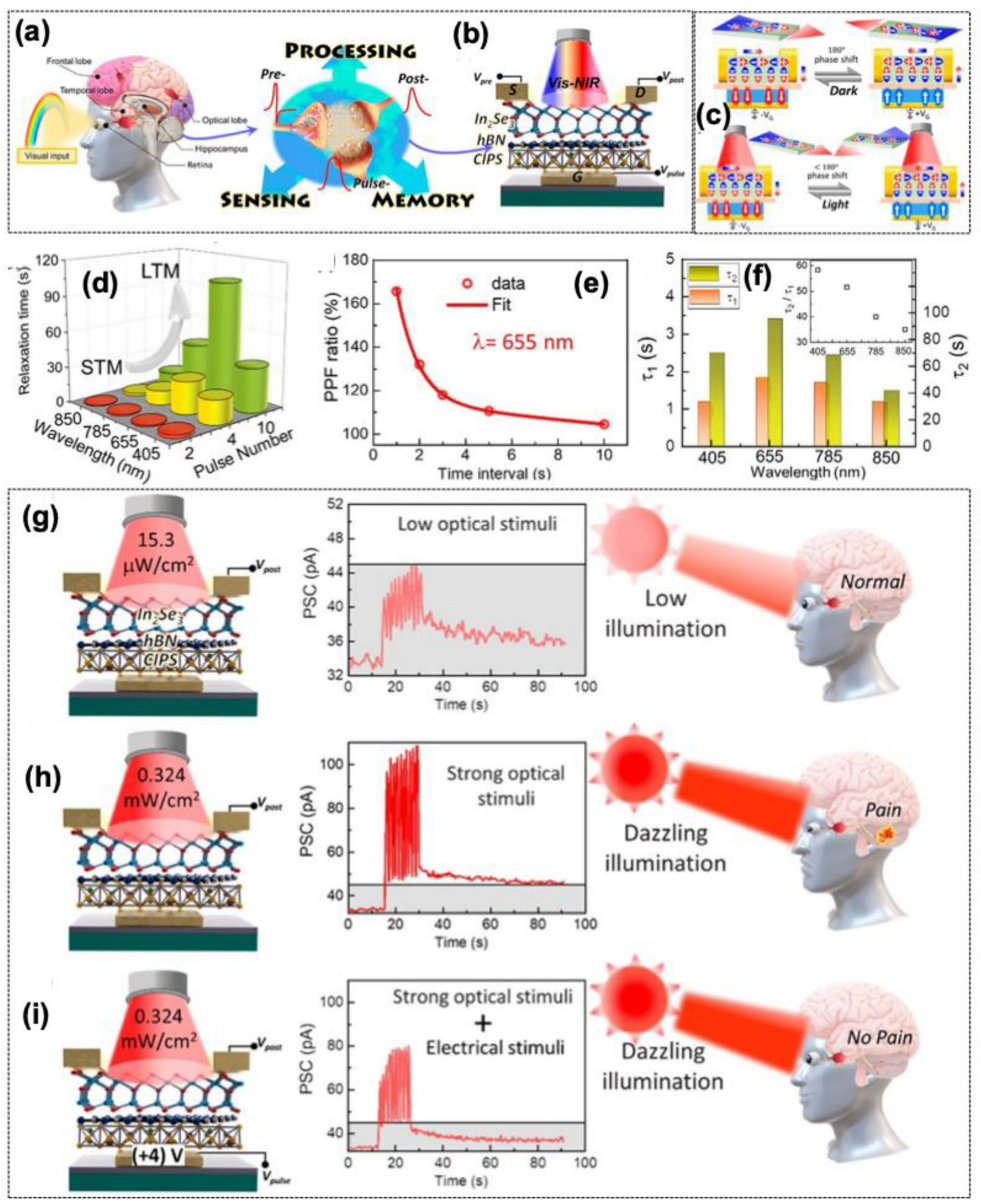
(a) The schematic illustration of the biological visual transmission under action potential, (b) Device structure diagram of the vdWH FeFET (CIPS/hBN/α‐In_2_Se_3_), (c) Schematic demonstration of the dipole polarizations under light (λ = 655 nm) conditions, (d) STM to LTM transition for all wavelengths (405 nm, 655 nm, 785 nm, 850 nm), (e) PPF ratio as a function of time interval for the excitation wavelength of 655 nm, (f) The relaxation time constant (τ_1_ and τ_2_) from the decay curves for different wavelengths, (g) light adaption test under low light illumination (15.3 mW/cm^2^). The current response under low light illumination is below the threshold (<45 pA) value, which could not cause injury to biological eyes, (h) under strong light illumination of 0.324 mW/cm^2^), the saturated current value exceeding the threshold level, which may damage the cells of the eyes, and (i) The artificial retina can adapt to this strong light through the modulation of positive gate voltage, and the saturated current values fall below the threshold level. Reproduced with permission [119]. 2026, American Chemical Society.

Das et al. [119] presented the development of a retina‐inspired artificial visual system based on a vdW heterostructure (vdWH) ferroelectric field effect transistor (FeFET), integrating CuInP_2_S_6_ and *α*‐In_2_Se_3_ layered materials as shown in Figure 14a,b. The vdWH FeFET platform enables in‐memory computing for artificial visual system, where ferroelectric polarization directly modulates channel conductance for synaptic functionality. The utilization of vdWH stacking reduces lattice mismatch constraints, supporting clean heterointerfaces compared to conventional oxide ferroelectrics. The device performance was mainly on the bidirectional locking of in‐plane and out‐of‐plane polarizations within the ferroelectric *α*‐In_2_Se_3_ layer, modulated by electrical and optical stimuli shown in Figure 14c. The device shows operation in the broad wavelength range of 405–850 nm and able to detect incident light intensities light intensities as low as low as 0.03 mW cm^−2^. In response to optical stimuli, the device exhibits STM and LTM transitions as shown in Figure 14d, with the ability to retain information beyond 2400 s by repeated electrical pulse stimulation simulating Pavlov's experiments. The device achieved a recognition accuracy of 92.5% on the MNIST dataset, indicating high suitability for pattern recognition tasks with a PPF ratio of approximately 170%, suggesting an effective temporal summation mechanism as shown in Figure 14e. Decay times for the conductance after stimuli ranged from τ_1_ ≈ 1–10 s (rapid relaxation) and τ_2_ ≈ 100–1000 s (slower decay), depending on operation parameters, illustrating a multi‐timescale memory process shown in Figure 14f. Neuromorphic performance, encapsulated by high synaptic plasticity under different stimulus conditions with PPF ratios and STDP‐like processes observed, indicating strong temporal and spatial learning capabilities.

The device successfully mimicked complex visual functions with 92.5% pattern recognition accuracy at a wide‐range wavelength sensitivity of 405–850 nm covering the visible spectrum and near infrared, allowing versatile detection of various light sources. Light adaptation behavior akin to biological iris reflexes, with responses to light intensities ranging from 15.3 µW cm^−2^ at 655 nm under low illumination, matching the way of biological eye adjusts to ambient light depicted in Figure 14g–i. The device also demonstrates optical logic operations for low‐power, multifunctional sensing and processing in artificial retinas.

**FIGURE 15:**
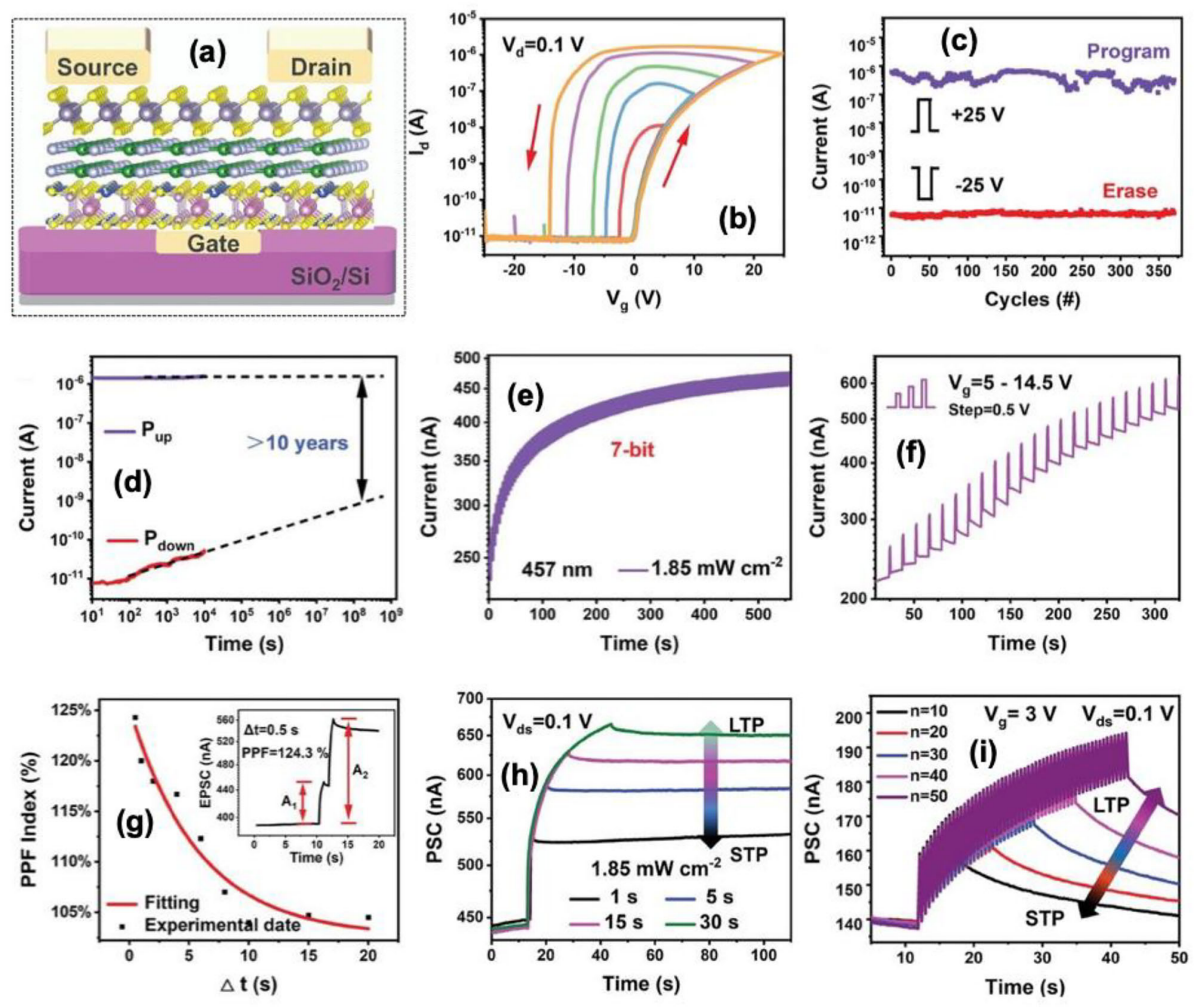
(a) Schematic representation of the three‐terminal vdWH Fe‐FET device, (b) transfer characteristics under different gate voltage scanning ranges (from ±5 to ±25 V, step: 5 V; V_ds_ = 0.1 V), (c) endurance characteristics of the fabricated device for over 350 P/E cycles, (d) retention characteristics of HRS (P_down_) and LRS (Pup) for the device, (e) Multilevel memory characteristics under periodic light pulses (457 nm, 1.85 mW cm^−2^, width: 1.5 s), (f) current response under different electrical pulse amplitudes (from 5 to 14.5 V, step: 0.5 V, width: 0.1 s) at read voltage of 0.1 V, (g) PPF index as a function of the pulse interval time (∆t) with a light intensity of 1.85 mW cm^−2^.The inset represents the PPF of two successive presynaptic spikes with ∆t of 0.5 s. The STP to LTP transition is achieved by (h) increasing the duration of light pulses, and (i) increasing the number of gate voltage pulses. Reproduced with permission under Creative Commons CC BY [46]. 2026, John Wiley and Sons.

Wang et al. [46] further described a novel full vdWH FeFET fabricated from layered SnS_2_, hexagonal boron nitride (h‐BN), and CIPS as shown in Figure 15a. The device demonstrated an ON/OFF current ratio exceeding 10^5,^ ensuring excellent current modulation crucial for reliable electronic operations. The heterostructure showed a retention time of more than 10^4^ s and cycle endurance exceeding 350 switching cycles, indicating robust and stable non‐volatile memory capabilities depicted in Figure 15b–d. The device supported 128 multilevel current states corresponding to a 7‐bit memory resolution crucial for high‐density information storage, shown in Figure 15e. The current response under different electrical pulse amplitudes (from 5 to 14.5 V, step: 0.5 V, width: 0.1 s) at a read voltage of 0.1 V is shown in Figure 15f. The full vdWH FeFET device successfully emulated key biological synaptic behavior, with the PPF index reaching a maximum of approximately 124% at a pulse interval (*Δt*) of 0.5 s, as shown in Figure 15g. The PPF index decreased exponentially with increasing *Δt*, following a double‐exponential decay fit, which is consistent with biological synapses. The device performed transient conductance changes with decay times on the order of hundreds of milliseconds to seconds, which can be modulated via light or electrical stimuli to mimic the rapid, reversible plasticity seen in the natural synapse. Persistent conductance enhancement was achieved after specific stimulation protocols, with conductance increases reaching up to 4.5 times the initial baseline. The transition from STP to LTP could be reliably induced by extending the duration or intensity of optical and electrical pulses, such as ±5 V, with a duration up to 10 s. The conductance could be decreased to below 20% of its maximum value through electrical depression stimuli, replicating the synaptic weakening mechanism as shown in Figure 15h,i.

The device performance exhibited beyond single‐device demonstrations by explicitly targeting an artificial vision system architecture using ferroelectric vdWH stacks, paving a pathway for integrating sensing, memory and computation without a von‐Neumann pipeline. However, this approach is still limited by vdWH assembly scalability, which is a severe bottleneck for wafer‐scale array manufacturing and uniformity.

**FIGURE 16:**
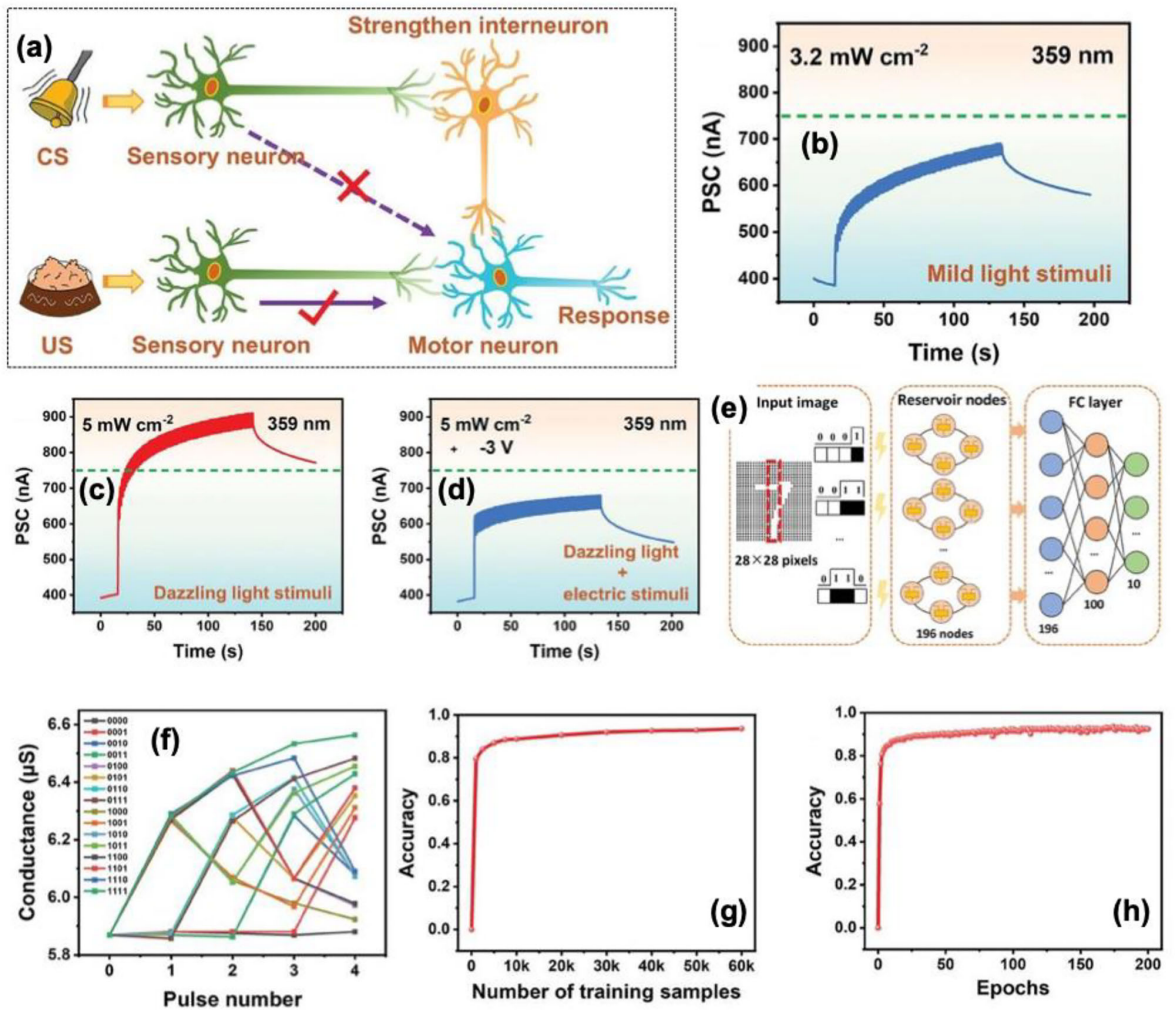
(a) Schematic illustration of the neural circuitry for associative learning. The current response (b) under a mild light stimulation, the PSC is below the threshold and does not cause eye damage, (c) under dazzling light stimulation, the PSC exceeds the threshold and causes damage to the eyes, (d) Adaptation behavior for the dazzling light stimulation by applying a negative voltage pulse of −3 V at a read voltage of 0.1 V, leading to a photocurrent below the threshold, (e) architecture and flowchart of RC for handwritten digital recognition, (f) 16 distinct conductance states with valid time series encodings ranging from 0000 to 1111, (g) Relationship between the image recognition accuracy and number of training images. (h) The recognition accuracy as the number of training iterations increased. Reproduced with permission under Creative Commons CC BY [46]. 2026, John Wiley and Sons.

Schematic illustration of the neural circuitry for associative learning shown in Figure 16a. The light adaptation behavior for the mild, dazzling, and combination of dazzling and electrical light stimulation by applying a negative voltage pulse and the corresponding current is shown in Figure 16b–d.

The integrated sensing‐memory‐computing device successfully demonstrated artificial vision functionalities as shown in the Figure 16e, by utilizing reservoir computing (RC) based on the optoelectronic features. The system achieved a recognition accuracy of 93.62% on the MNIST dataset. 16 distinct conductance states with valid time series encodings ranging from 0000 to 1111 are presented in Figure 16f. The accuracy improved markedly with increased training data from 79.65% with 1000 images to 93.62% with 60 000 images. Additionally, training iterations further refined the performance, with accuracy plateauing at 93.62% after sufficient training shown in Figure 16g,h. Simulated associative learning demonstrated the potential of the system for complex cognitive processing of visual tasks. However, further enhancement is necessary for commercial viability and the relatively high gate voltage (±25 V) for ferroelectric switching, which could pose energy efficiency challenges. Further investigation is needed for the platform must demonstrate array‐level yield and stable reliable performances under repeated electrical and optical stimuli and CMOS BEOL‐compatible process line for future practical deployment.

**FIGURE 17:**
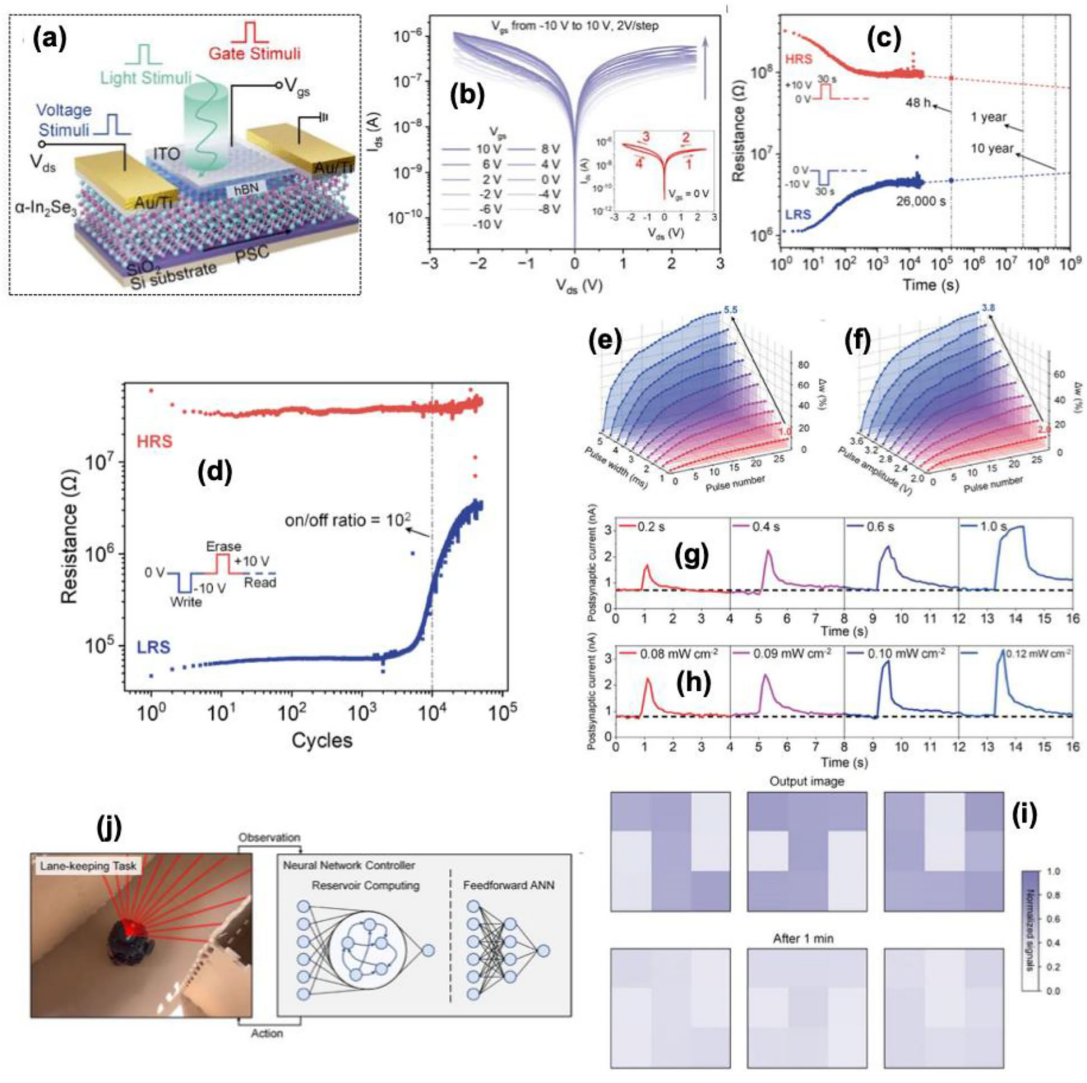
(a) Schematic of the single‐transistor PCIM synapse on a SiO_2_/Si substrate, (b) output characteristics of the fabricated device with consecutive measurements over a V_ds_ range (−2.5 to 2.5 V), at different V_gs_ bias (−10 to 10 V) with 2 V step, showing wide gate tunable conductance states. The inset shows the results of ten consecutive I_ds_−V_ds_ sweeps measured without gate bias, (c) retention characteristics, with sufficient P/E pulses (±10 V, 30 s, and read at V_gs_ = 0 V, V_ds_ = 1 V), (d) endurance of α‐In_2_Se_3_ FeSFET PCIM (±10 V, 400 ms and read at V_gs_ = 0 V, V_ds_ = 1 V), representing ON/OFF ratio of 10^2^, (e) The percentage change of conductance (Δ*W*) under 30 consecutive *V*_ds_ voltage pulses (amplitude of 3 V) with different voltage widths (1.0–5.5 ms in 0.5 ms steps), (f) The percentage change of conductance (Δ*W*) under 30 consecutive voltage pulses (width of 3.5 ms) with different voltage amplitudes (0.2 V steps from 2.0 to 3.8 V, (g) Higher channel current with increased illumination time (0.2, 0.4, 0.6, and 1.0 s, 0.08 mW cm^−2^ fixed light power), (h) PSC when light pulses (0.08, 0.09, 0.10, and 0.12 mW cm^−2^, 0.4 s fixed illumination time) were applied to the device, exhibiting higher photocurrent with increased light power, (i) Current map of the image memory of the letters Z, J, and U with a 3 × 3 array. The letter areas were stimulated with a light intensity of 0.16 mW cm^−2^ (pulse width, 1 s). (j) Schematic of the lane‐keeping task. The distance to obstacles is observed by the laser and input to the network. The output of the network is used to modify the steer angle of the car. Reproduced with permission under Creative Commons CC BY [120]. 2026, American Chemical Society.

Zhou et al. [120] demonstrated a ferroelectric synaptic transistor that employs *α*‐In_2_Se_3_ as the channel material with a layered hexagonal (2H) phase. The device architecture includes a ∼20 nm thick hBN dielectric layer and Ti/Au source and drain electrodes, shown in Figure 17a. The device exhibited a clear hysteresis window of 1.2 V with a high ON/OFF ratio exceeding 10^4^, enabling distinct conductance states suitable for memory and neuromorphic behavior depicted in Figure 17b. The device demonstrates a multimodal 2D ferroelectric transistor that integrates perception and in‐memory computing, moving toward multimodal neuromorphic perception rather than only optical or only electrical synapses. The demonstration represents a clear direction toward state‐of‐the‐art future neuromorphic hardware that must fuse multiple sensory inputs including vision, tactile, optical and electrical stimuli. The paper is an example of multimodal fusion at device level, rather than system‐level software fusion.

The device switching performance was validated by 10 000 endurance cycles with less than 5% variation and long‐term data retention of more than 48 h with negligible decay, indicating excellent non‐volatile switching stability as shown in Figure 17c,d. The LTP and LTD were achieved by high‐frequency electrical pulses with 100 Hz (LTP) and 1 Hz (LTD), with a conductance change of approximately 200% (LTP) and a reduction by 80% (LTD). The percentage change of conductance (ΔW) under 30 consecutive Vds voltage pulses (amplitude of 3 V) with different voltage widths (1.0–5.5 ms in 0.5 ms steps) and the percentage change of conductance (ΔW) under 30 consecutive voltage pulses (width of 3.5 ms) with different voltage amplitudes (0.2 V steps from 2.0 to 3.8 V shown in Figure 17e,f. The optical responses were recorded with 532 nm laser illumination at a fixed light intensity of 0.08 mW cm^−2^. The device exhibits significant photo response, reaching photocurrent levels of approximately 50 nA (initial) and rising to over 1 µA after a 0.4 s illumination period. Increasing illumination duration up to 1 s and light intensities from 0.08 to 0.12 mW cm^−2^ enhances conductance levels and trap filling effects, facilitating more persistent memory states as shown in Figure 17g,h.

The system integrates the device with a reservoir computing (RC) framework for real‐time obstacle detection and lane‐keeping tasks in robotic applications. Experimental results show adaptive control with computed linear and angular velocities matching trajectories obtained from real‐world robotic experiments.

The system‐level integration with a 3 × 3 array size was fabricated, in which each cell is capable of sensing and storing optical images. Exposure text with optical mask (letters Z, J, U) at a light intensity of 0.16 mW cm^−2^ and PW of 1 s resulted in the measurable current increases, which persisted over 1 h after light removal, as shown in Figure 17i. The memory states were stable with minimal fluctuation of < 3% over this period, which determines the effectiveness of combined optical sensing and memorization. The incorporation of transistor‐based platforms are more favorable for dense array than two‐terminal devices because they allow better controllability and reduced sneak paths.

However, scaling and device arrays beyond the demonstrated 3 × 3 remain challenging; uniformity and fabrication precision need more modification. The current switching speed of ∼100 µs may limit the application requiring ultrafast operations. Future work should be focused on optimizing device architecture for high‐speed and low‐voltage operation, stable analog weight update larger array integration validation, and environmental robustness under repeated multimodal cycling for practical neuromorphic system deployment.

**FIGURE 18:**
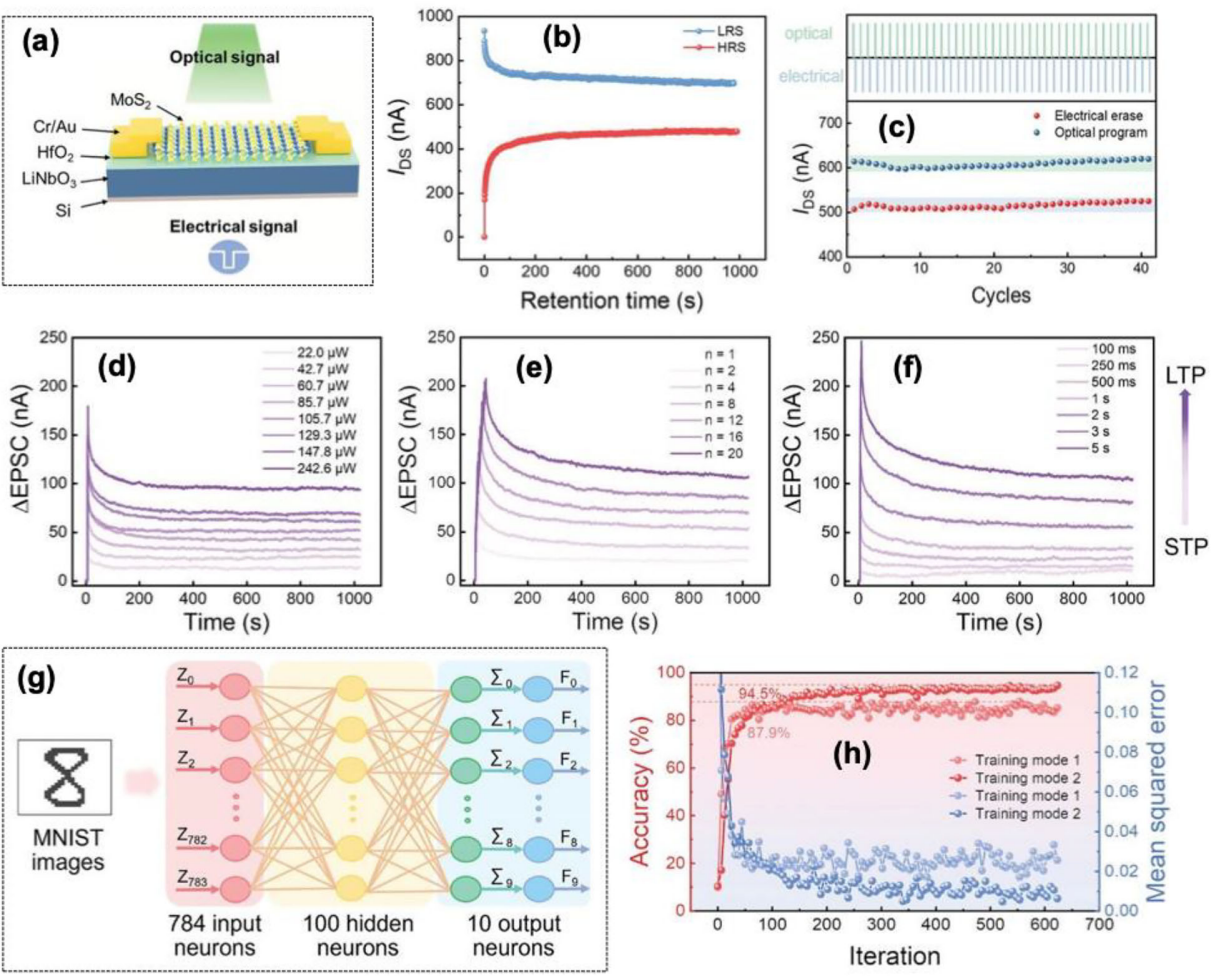
(a) Schematic illustration of 2D ferroelectric optoelectronic transistor driven by optical and electrical signals, (b) The retention characteristics of LRS and HRS for 10^3^ s after optical/electrical switching, respectively, (c) endurance characteristics of optoelectronic memory switching behavior, STP/LTP transition, (d) ∆EPSC versus pulse intensity from 22 to 242.6 µW of (e), versus pulse number from 1 to 20 of (f), versus pulse width from 100 ms to 5 s, (g) A schematic illustration of the simulated artificial optoelectronic network for image recognition tasks, where 28 × 28 pixels image of representative MNIST handwritten images are used to train and test the network, and (h) Accuracy and mean squared error of digital image recognition during the training epochs. Reproduced with permission [101]. 2026, John Wiley and Sons.

Wang et al. [101] further explored the controllable and compact 2D optoelectronic transistor architecture shown in Figure 18a based on ferroelectric lithium niobate (LiNbO_3_) to simulate the neuromorphic visual system, which permits the integration of reconfigurable memory–sensing–processing and logical operations under an operating voltage of ∼1.5 V. The photoelectron trapping effect at the MoS_2_/HfO_2_ interface and the strong spontaneous polarization of LiNbO_3_ resulted in the reliable and highly controllable opto‐electrical memory characteristics. This paper emphasizes feasible CMOS compatible low voltage operation compared to vdW ferroelectric systems.

The device supports more than 16 discrete conductance levels, approximately equivalent to 4‐bit data storage for multilevel storage capability with 10^3^ s of data retention capability, confirming the non‐volatile memory characteristics shown in Figure 18b. The endurance characteristics of optoelectronic memory switching behavior are shown in Figure 18c. The optical logic operation, specifically ‘AND’ and ‘OR’, was utilizing dual‐wavelength inputs of 532 and 808 nm. The input signals are represented as ‘light open’ for logic “0” and ‘light closed’ for logic “1.” The device completed logic operation within a small bias voltage of ∼1.5 V. STP/LTP transition with different intensity, pulse number, and time is shown in Figure 18d–f. The device‐based neural network depicted in Figure 18g achieved a recognition accuracy of approximately 94.5% after 620 training iterations, recognizing hand‐written digits in the MNIST dataset. The normalized conductance maps for 10 output neurons show consistent updates after 600 iterations. A significant enhancement over a lower accuracy of 87.9% achieved in a different training mode, highlighting the importance of conductance linearity and stability shown in Figure 18h. System‐level verification was utilized 3 × 3 array emulate biological learning and memorization behaviors, capable of retaining learned topics for up to 10 min after laser removal under high‐intensity learning conditions. Conductance updates with LTP and LTD can be performed with a 5 s PW and intensity of 64 mW cm^−2^, and the repeatability. The proposed device stack incorporated with the mainstream CMOS compatible HfO_2_ including robust ferroelectric functionality, make it technological relevant for future technological nodes.

The main limitation arises with long‐term stability of low retention of 10^3^ s and endurance, which limits the practical application in the neuromorphic hardware. The discrete conductance states (∼16) though sufficient for synaptic weight update, but more extensive device variation (illumination area) can result in inconsistency in the weight update. The device operation voltage is ∼1.5 V, is CMOS‐compatible. The power efficiency during large‐scale array level and the operational speed, particularly for uniform ferroelectric switching and logic processing, likely depend on the PW (5 s) is relatively slow for faster dynamics that are desirable for high‐speed wafer‐scale integration and yield statistics for in‐memory computation.

**FIGURE 19:**
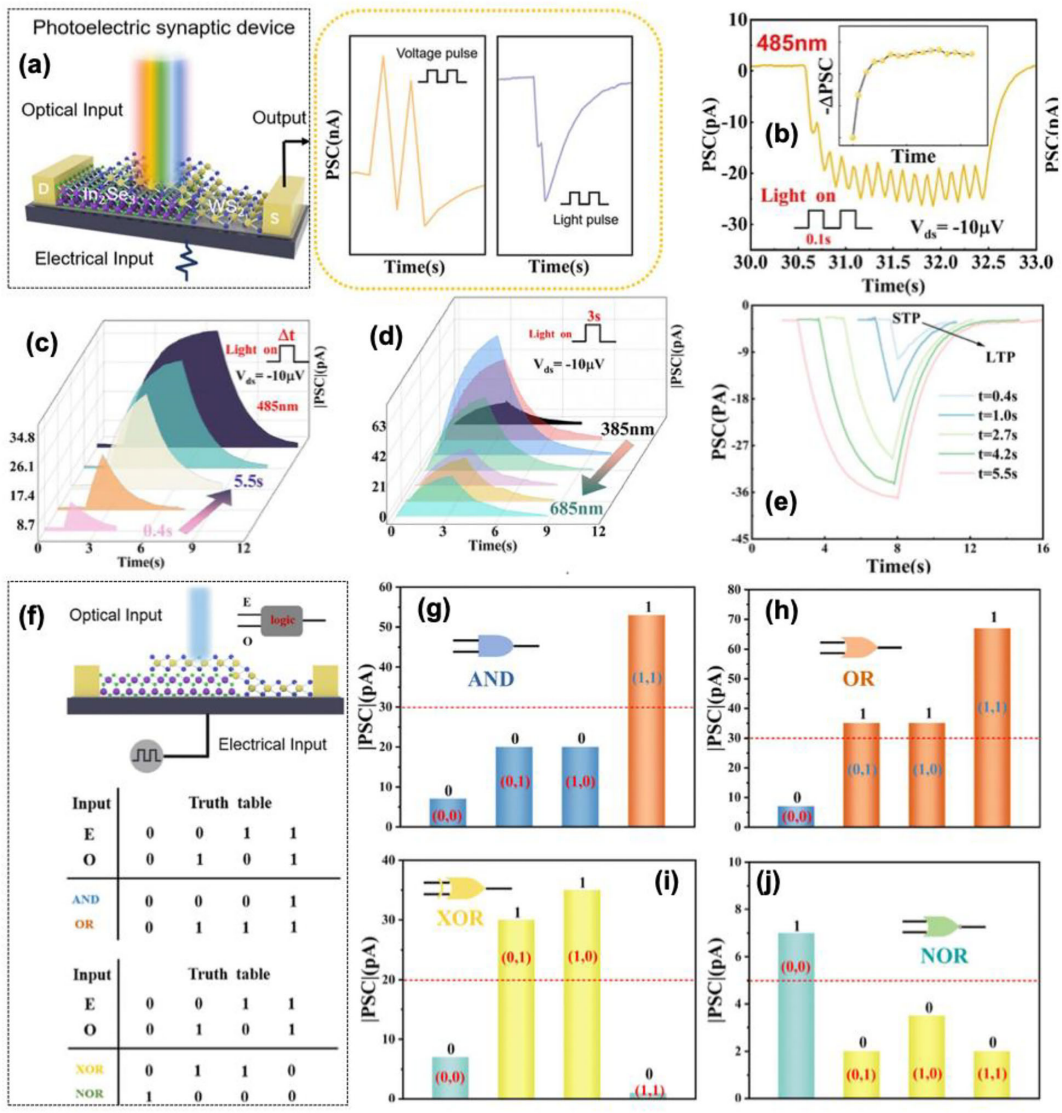
(a) The schematic representation of the device architecture along with the current response under different pulses, (b) PSC with constant light pulses (485 nm, *V*_ds_ = −10 µV). The inset shows the change of ΔPSC over time, PSC response measured with (c) light stimulation durations (485 nm, *V*_ds_ = −10 µV), (d) light stimulation durations (385 to 685 nm, *V*_ds_ = −10 µV), (e) PSC measured with different light stimulation durations, (f) The schematic diagram and truth tables of the logic gate (AND, OR, XOR, and NOR) device based on the WS_2_/In_2_Se_3_ vdW memtransistor, (g) AND logic operation result with the inputs of *V*_G_ = −15 V and illumination of 415 nm, (h) OR logic operation result with the inputs of *V*_G_ = −20 V and illumination of 435 nm, (i) XOR logic operation with *V*_G_ = 20 V and illumination of 400 nm, (j) NOR logic operation with *V*_G_ = −3 V and illumination of 373 nm. Reproduced with permission [102]. 2026, American Chemical Society.

**FIGURE 20:**
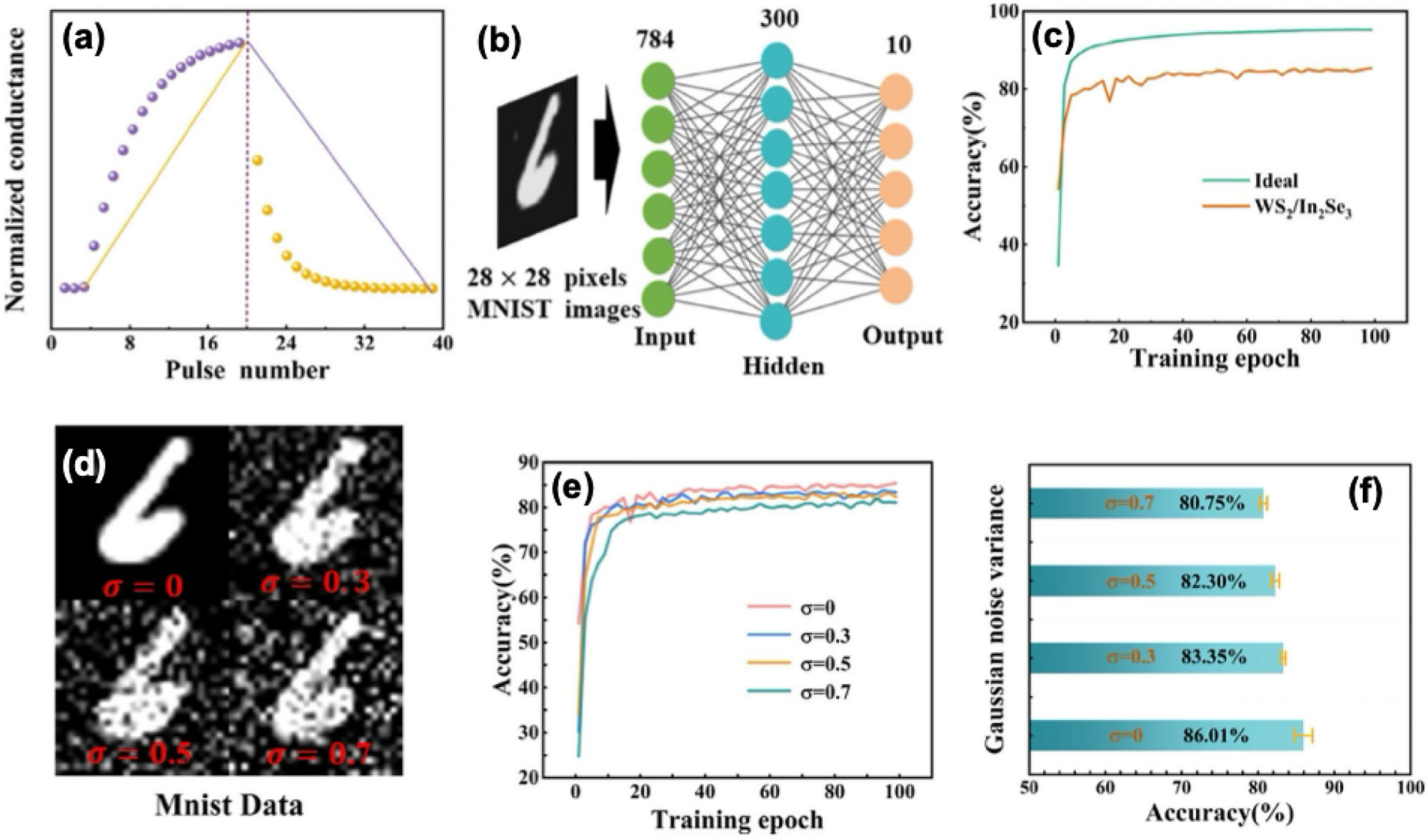
(a) The potentiation and depression conductance of the WS_2_/In_2_Se_3_ optoelectronic memtransistor. (b) Schematic of the three‐layer neural network used for MNIST image recognition. (c) Comparison of recognition rates between the ideal device and the WS_2_/In_2_Se_3_ optoelectronic synaptic device. (d) MNIST data with different levels of Gaussian noise. (e) Recognition rates of the WS_2_/In_2_Se_3_ optoelectronic synaptic device under different standard deviations of Gaussian noise. (f) The corresponding accuracy values after 100 training epochs under multiple calculations. Reproduced with permission [102]. 2026, American Chemical Society.

Ma et al. [102] demonstrated a vertical WS_2_/In_2_Se_2_ vdWH memtransistor to emulate key synaptic behavior and perform the logic operation through combined electrical and optical stimuli shown in Figure 19a. The device enabling optoelectronic synaptic behavior with ultralow energy per optical spike, benefiting from vertical transport and ferroelectric polarization modulation. The device exhibited attojoule (aJ)‐level energy consumption, which is among the lowest reported for optoelectronic synaptic devices. Electrical inputs were controlled via gate voltages typically SET at 0 V (logic 0) and −15 or −20 V (logic 1) with a pulse duration of 10 ms. Optical stimuli involved light pulses at wavelengths such as 485 and 385 nm, serving as optical “1” inputs, with darkness representing “0.” PSC with constant light pulses (485 nm, *V*_ds_ = −10 µV). The inset shows the change of ΔPSC over time, PSC response measured with light stimulation durations (485 nm, *V*_ds_ = −10 µV), light stimulation durations (385 to 685 nm, *V*_ds_ = −10 µV), and PSC measured with different light stimulation durations shown in Figure 19b–e. For instance, the AND logic functions were demonstrated by applying −15 V electrical bias and optical excitation at 415 nm, with an output current exceeding 30 pA designated as logical “1.” Similar configurations enabled the other logic gates with tailored input conditions shown in Figure 19f–j. The optical pulses, delivered for approximately 10 ms, corresponded to optical power densities in the range of 10–100 µW cm^−2^, depending on wavelength. The energy consumption for each spike was remarkably low, measured at approximately 7.7 aJ, calculated using a typical read voltage of 10 µV and peak currents in the picoampere (pA) range during stimulation. The device's conductance state, represented by the drain–source currents (*I_D_*), ranged from tens to hundreds of picoamperes, enabling both STP and LTP as shown in Figure 20a. These conductance states were stable for extended periods of more than 10^4^ s, confirming non‐volatile memory capability.

The neuromorphic performance of the device was achieved with an image recognition accuracy of 85.41% on the MNIST dataset with a single‐layer neural network, illustrated in Figure 20b,c. The recognition accuracy was maintained at 83.35% to 80.75% with the introduction of the Gaussian noise with a standard deviation (σ) of 0.3 to 0.7, respectively, demonstrating the robust noise tolerance as shown in Figure 20d–f. Logic operations such as AND, OR, XOR, and NOR were realized by combining electrical gate voltages of −15, 0, or −20 V with optical illumination at specific wavelengths 415 nm, 435 nm, 400 nm, 373 nm. The vertical heterostructure geometry can simultaneously improve synaptic response strength and energy efficiency, which is relevant for future low‐power in‐sensor neuromorphic vision nodes.

Despite promising performance, the electrical gate voltages required for logic operations are relatively high (−15 to −20 V), which pose integration challenges for scalable circuits. Additionally, the switching speed (10 ms) is slower than biological synapses (∼1 ms), limiting real‐time processing capabilities. Achieving faster response times remains an essential goal. Device variability and fabrication reproducibility such as mechanically stacked or dry‐transferred vdWH for scalable wafer‐level arrays should be improved. Furthermore, expanding the spectral responses range and optimizing power efficiency across different wavelengths including stability under repeated combined electrical and optical cycles will enhance the device's versatility for vision and sensing applications.

**FIGURE 21:**
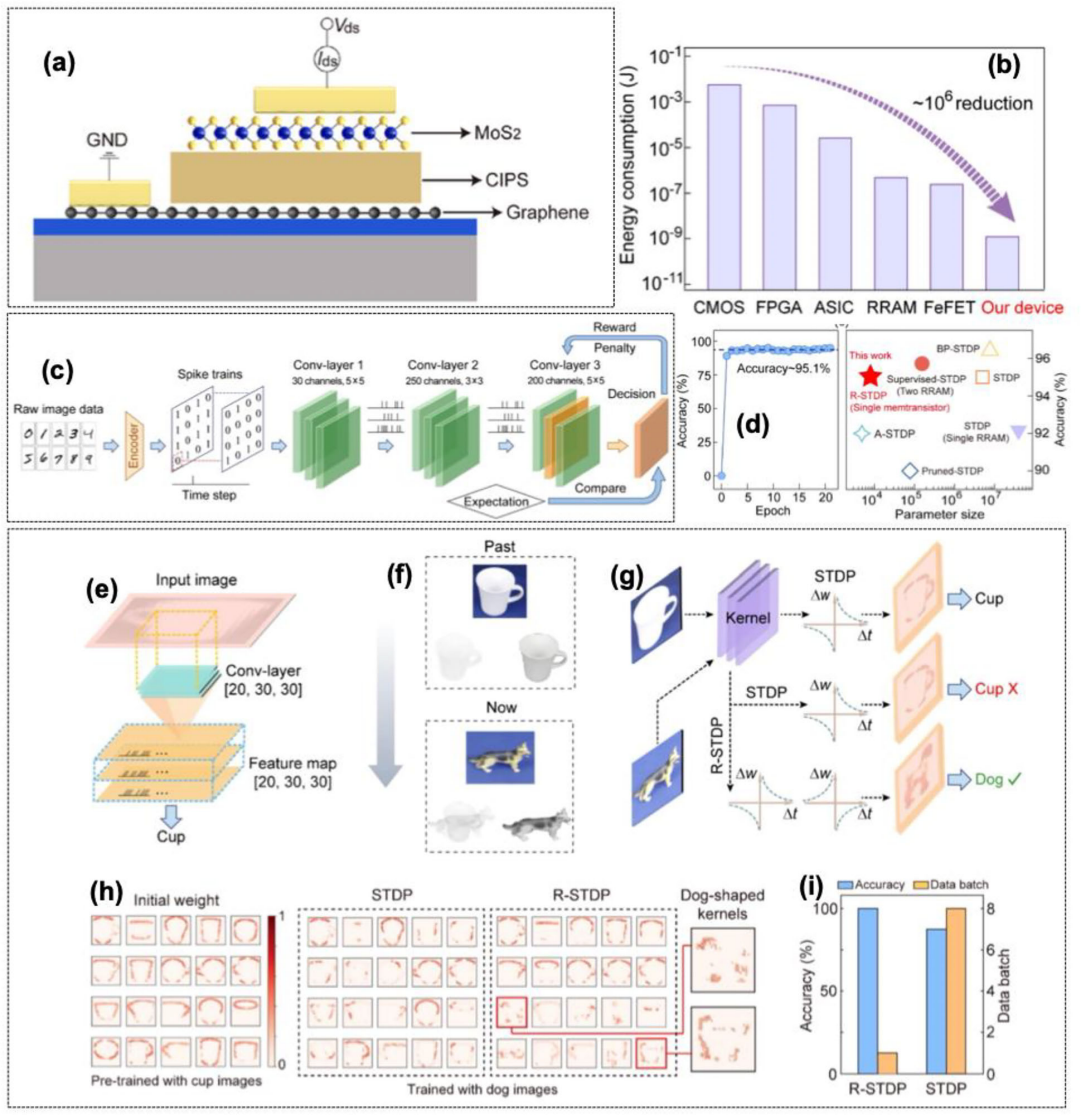
(a) Schematic diagram of vertical MoS_2_/CIPS/graphene heterostructure, (b) Comparison of the energy consumption in implementing R‐STDP by using different hardware devices, (c) The overall structure of MNIST recognition network trained with R‐STDP, (d) Simulated recognition accuracy in terms of training epochs and SNN works focusing on STDP‐based training, (e) The structure of the single‐layer network used for the few‐shot learning task. (f) The simplified schematic illustration of the few‐shot learning process, in which the cup is learned first, followed by the learning of the dog. (g) Learning process of the task in detail. (h) Weight distributions of the STDP and R‐STDP networks after training. (i) Comparison of the inference accuracies and learning speed in the few‐shot learning task for R‐STDP and STDP trained networks. Reproduced with permission [121]. 2026, Elsevier.

Cao et al. [121] present a 2D ferroelectric memtransistor based on CIPS vdWH enabling reconfigurable learning rule designed as shown in Figure 21a to emulate reward‐modulated spike‐time‐dependent plasticity (R‐STDP) within a single device architecture, a fundamental learning rule, a key step toward hardware spiking neural network (SNN) intelligence. This approach harnesses the tunable Schottky barrier effect at the ferroelectric interface, facilitating electronic emulation of complex neuro‐modulatory influences akin to dopamine signaling.

The device consumes approximately 1.3 nJ per switching cycle, representing a reduction of about 10^6^ times relative to the neuromorphic circuits, demonstrating its suitability for large‐scale energy‐efficient neuromorphic systems in Figure 21b. Polarization reversal is achieved with voltages within ±5 V, enabling low‐power control with a high memory window. HRS to LRS ratio increased from ∼1.1 × 10^2^ at initial states to exceeding 1.8 × 10^5^ with increased operating voltage. The endurance of the device exhibited more than 100 switching cycles, indicated the reliability and endurance for learning applications.

The memtransistor accurately emulates biological synaptic behaviors, notably STDP and reward‐modulated STDP (R‐STDP). The device's conductance change follows spike‐timing relationships, with stable potentiation (weight increase) and depression (weight decrease), adjustable through the timing and voltage pulsing scheme. The transition between STDP and anti‐STDP modes is achieved via gate voltage tuning, effectively capturing neuro‐modulatory effects and enabling reward‐based learning.

The neural network performance was utilized by integrating a SNN training with S‐STDP, shown in Figure 21c. The system achieved an accuracy of 95.2% on the MNIST dataset with only 8000 stimuli, illustrating high learning capacity with minimal hardware resources, as shown in Figure 21d. The device demonstrated fast convergence in few‐shot learning scenarios, requiring just a single batch of 100 samples for full inference, indicating excellent adaptability and learning efficiency. The structure of the single‐layer network used for the few‐shot learning task and its simplified schematic illustration of the few‐shot learning process, in which the cup is learned first, followed by the learning of the dog, is shown in Figure 21e,f. Learning process of the task in detail and the weight distributions of the STDP and R‐STDP networks after training are shown in Figure 21g,h. Last, the comparison of the inference accuracies and learning speed in the few‐shot learning task for R‐STDP and STDP trained networks is shown in Figure 21i.

The device's neuromorphic visual application exhibited a robotic control task employing the R‐STDP‐trained SNN. The system exhibited an 85.5% success rate in tracking both static and moving targets, highlighting its robustness and ability to adapt to dynamic environments in real‐time applications including significant comparison with a CMOS‐based implementations that requires ∼50 000 transistors. The system's agility underscores its compactness advantage of device‐native learning and potential for deployment in autonomous robotics.

However, the device marks a substantial advancement in neuromorphic hardware. The current device demonstrated a single prototype device with device‐to‐device uniformity. Large‐scale fabrication of uniform ferroelectric 2D heterostructures remains challenging, which could impact the consistency of synaptic behavior in mass production. The switching speed and power consumption per operation are also constrained by ferroelectric polarization dynamics, which may limit real‐time learning speeds in high‐frequency parallel arrays for neuromorphic systems. Although the device itself consumes minimal energy, the overall power efficiency depends on the peripheral circuits for signal processing, which may introduce additional energy overheads that need extensive study for practical applications.

## 6 Practical Challenges, Realistic Reliability Concerns and Future Opportunities

### 6.1 Practical Challenges, Array Uniformity and Scalability Concern

Despite the rapid progress of 2D ferroelectric memories toward integrate artificial vision and neuromorphic computing, translating laboratory demonstrations into manufacturable, high‐yield arrays remain non‐trivial. Recent studies have shown that optoelectronic and polarization‐controlled plasticity can be achieved through diverse material/device platforms, including optical‐electrical coordinately modulated 2D ferroelectric RP perovskite memristor for artificial vision [46], multisensory ferroelectric semiconductor synapses for multimodal neuromorphic perception [110], and light‐enhanced switching in in‐plane ferroelectric NbOI_2_ memristors [111]. Beyond single‐device synapses, several works have explicitly targeted artificial vision architectures by integrating in‐memory sensing and computing in full vdWs heterostructures [100, 119], while multimodal 2D ferroelectric transistors further extend this paradigm toward integrated perception and computing‐in‐memory for multi‐stimulus tasks [120]. Heterostructure engineering further improves functional robustness, as exemplified by Bi_2_Te_3_/SnSe hetero‐memristors targeting stable bionic‐visual systems [112] and Bi‐doped SnSe ferroelectric memristors enabling all‐in‐one sensing‐memory‐computing [113]. Importantly CMOS‐relevant material stacks are also emerging, such as LiNbO_3_/HfO_2_/MoS_2_ synaptic transistor platform demonstrates reconfigurable sensing‐memory‐processing and logical integration, highlighting a more manufacturing oriented pathway compared with purely exfoliated vdW assemblies [101]. At the transistor level, polarization modulated multi‐mode optoelectronic synaptic transistors based in *α*‐In_2_Se_3_/SnS_2_ heterojunctions integrate sensing, memory, logic (AND/OR) and optical wireless communication, however, they still require a large memory window (∼76 V), endurance>800 cycles and retention >10^4^ s, highlighting both integration progress and reliability constraints [77].

From the manufacturability perspective, the dominant bottleneck is the wafer‐scale 2D ferroelectric synthesis with tight control over thickness, defect density, and ferroelectric domain distribution. Such non‐uniformities translate into device‐to‐device variations in coercive voltage, switching thresholds, analog weight‐update linearity and photoresponse, which directly degrade learning accuracy in a large crossbar or retina‐inspired arrays. Furthermore, the interface/contact sensitivity of vdW heterostructures introduces additional variability via trap‐assisted transport, Schottky barrier fluctuations, and local polarization pinning. These effects become more severe under repeated optical excitation, where photo‐generated carriers and interfacial charge trapping can ensure drift in synaptic states and accelerate fatigue.

To overcome these scalability barriers, ongoing efforts are increasingly focusing on scalable material synthesis routes (CVD/MBE/epitaxy) to improve crystallinity, reduce defect density, and enable polarization or domain control across large areas [60, 61, 62, 63]. This is particularly critical for sliding ferroelectric semiconductors where mechanically exfoliated flakes and limited ferroelectric domain size remain major bottleneck for practical issues [64]. Interface engineering and contact optimization to suppress trap‐dominated switching variability and improve the reproducibility of polarization controlled Schottky barriers in ferroelectric semiconductor synapses relying on graphene/*α*‐In_2_Se_3_/graphene junctions and heterointerfaces. Encapsulation and passivation strategies to mitigate oxygen or moisture‐induced degradation and stabilize optoelectronic responses, which remains a non‐trivial issue for ultrathin vdW ferroelectric devices and light‐modulated memories, particularly under repeated illumination cycling and long‐term operation [77, 110, 111]. Device and circuit codesign approaches, such as array level in‐sensor computing demonstrations and algorithm‐hardware co‐optimization, which explicitly address residual non‐uniformity and enable practical pattern recognition under non‐ideal device statistics [110, 112]. Collectively, these strategies will be essential for transforming 2D ferroelectric optoelectronic memories from proof‐of‐concept devices into scalable, reliable and system‐level viable artificial vision and neuromorphic computing platforms.

### 6.2 Reliability Under Realistic Optical‐Electrical Operating Conditions

In addition to scalability, long‐term reliability under realistic operating conditions remains a decisive challenge for commercialization. In 2D ferroelectric optoelectronic synapses, repeated electrical programming can induce polarization fatigue, imprint and coercive‐voltage drift, while continuous or cyclic illumination introduces additional degradation channels via photo‐generated charge trapping at interfaces and contacts, which can gradually distort analog weight‐update linearity and retention. These reliability issues become amplified in large arrays, where small device‐to‐device drifts accumulate into substantial accuracy loss at the system level. Therefore, beyond reporting endurance and retention at the stand‐alone device level, future studies should increasingly quantify failure modes under combined optical‐electrical stress, elevated temperature and humidity exposure, and establish physics‐based mitigation strategies. Promising ongoing directions include trap‐suppressed interface engineering in ferroelectric semiconductor synapses and heterojunction transistors, stability‐oriented heterostructure design for bionic vision system and CMOS‐compatible stacks that can leverage mature encapsulation and BEOL reliability practices [64, 77, 101, 110]. In parallel, ultra‐low energy 2D vdW heterostructure memtransistors, such as, WS_2_/*α*‐In_2_Se_3_, highlight that energy efficiency can already approach attojoule‐level per optical spike, however, their present demonstration will rely on mechanically assembled dry‐transfer stacks on SiO_2_/Si and require relatively high gate biases for logic, underscoring the need for scalable epitaxial growth, low‐voltage electrostatics, and array‐level reproducibility for manufacturable neuromorphic hardware. Beyond sensory synapses, neuromodulation‐aware learning rules are also beginning to be realize at the device level, a vdW ferroelectric memtransistor was shown to reconfigure STDP and anti‐STDP within a single multi‐terminal device to implement reward‐modulated STDP (R‐STDP), achieving ∼1.3 nJ energy per event and demonstrating robotic recognition and tracking with SNN hardware characteristics [102, 121]. These emerging device‐level learning primitives further reinforce the necessity of reliability‐aware co‐design, where endurance, drift, and environment‐induced variability are evaluated together with learning accuracy convergence under realistic workloads.

### 6.3 Future Outlook and Opportunities

The rapid progress in 2D ferroelectric materials has opened a transformative pathway for the design of next‐generation optoelectronic vision sensors capable of emulating biological perception, performing in‐sensor information processing, and enabling real‐time neuromorphic functionalities. Research continues to reveal the fundamental advantages of van der Waals ferroelectrics, including switchable out‐of‐plane polarization, atomic‐level thickness control, tunable band alignment, and intimate coupling between ferroelectric and optoelectronic responses. The emergence of materials, such as α‐In_2_Se_3_, CIPS, MoS_2_‐based ferroelectric heterostructures, and hybrid ferroelectric‐semiconducting stacks, has demonstrated the potential to simultaneously integrate sensing, memory, and learning processes directly within the pixel plane, thereby challenging the longstanding dominance of traditional von‐Neumann architectures [85, 122]. These developments point toward a future where compact, energy‐efficient, and highly adaptive sensing systems can operate autonomously at the device level.

The application of 2D materials in optoelectronic vision sensors holds immense promise for next‐generation artificial visual systems. Their unique ferroelectric properties combined with excellent light‐matter interactions facilitate a highly sensitive, energy‐efficient, and flexible devices capable of advanced functionalities such as non‐volatile memory, broadband detection, and neuromorphic processing [83]. The atomically thin, mechanically flexible nature of these materials makes them particularly suitable for integration into conformal and portable systems, including bio‐inspired smart lenses, artificial retinas, wearable health monitors and soft robotic vision platforms. Recently reported 2D ferroelectric materials‐based optoelectronic memristor and memtransistor devices' performances and their application in the neuromorphic visual perception have been summarized in Table 1.

**TABLE 1:** Figure of merits of reported 2D ferroelectric materials based‐optoelectronic memristor and memtransistor devices along with their applications.

Device type	Functional materials	Memory window	Endurance	Recognition accuracy	Applications
Photodiode/homojunction Memristor [48]	P(VDF‐TrFE)/MoTe_2_/Graphene	−800 to +800 mA/W	>10^6^ cycles, 51 memory states	Patterns (array: 3 × 3) classification (‘L’, ‘⅃’, ‘T’)	Broadband optical response: 340–1310 nm
Ferroelectric FET [123]	α‐ In_2_Se_3_/ Graphene heterostructure	55 V	>10^6^ cycles	—	Broadband optical response: 406, 638, and 980 nm
Memristor [124]	ML Graphene/CIPS/Au	10^2^	—	—	Mimics human visual adaptation
Ferroelectric Transistor [125]	P(VDF‐TrFE)/ReS_2_	10^9^	—	89%	96% visual perception, noise suppression after preprocessing, blue (450 nm), green (530 nm), red (650 nm)
Ferroelectric Transistor [101]	MoS_2_/HfO_2_/LiNBO_3_	>10×	10^3^ s, 16 MLC states	94.5%	Dual wavelength logic (AND/ OR) at 532 nm, 808 nm, pixel‐wise logic‐based image preprocessing
Ferroelectric Transistor (vdW) [46]	SnS_2_/h‐BN/CIPS	10^5^	10^4^ s, 128 MLC states	93.62%	Retina‐like visual processing using reservoir computing (light adaptation, Pavlovian conditioning, dynamic PSC modulation)
Ferroelectric phototransistor [126]	MoS_2_/Ba_0.6_Sr_0.4_TiO_3_ (BST)	10^5^	10 h	95.87%	Broadband photodetection (400–1100 nm), In–memory logic gates (AND, OR, NOT, NAND, NOR)
Memristor [110]	Graphene/𝛼‐In_2_Se_3_/graphene	10^2^	40 ns *WRITE* speed, 12 MLC states	97%	Multisensory retinomorphic in‐sensor processor, broadband photodetection (340–940 nm)
Memristor (vdW ambipolar FeFET) [127]	Graphene/MoTe_2_/h‐BN/𝛼‐In_2_Se_3_/ graphene	10^2^	100 cycles	94.5%	Retinomorphic functions (optical weight update, light‐induced synaptic plasticity, and encoding of visual stimuli), broadband photodetection
MFMIS FeFET [128]	MoS_2_/h‐BN/Au/Cr/AlScN	10^5^	350 P/E cycles	93.62%	Optical decoding, brain‐like visual memory, and synaptic processing
FeFET (vdW) [98]	𝛼‐In_2_Se_3_/h‐BN/CIPS	10^4^	—	(array: 7 × 7) Pattern classification of ‘L’‐shape	Broadband photodetection (405–660 nm), dynamic adaptation functionalities of retina‐like behavior
Ferroelectric transistor (vdW) [129]	𝛼‐In_2_Se_3_/WSe_2_	∼10	25 MLC states	96%	Broadband photodetection (380–780 nm), noise suppression, and nonlinear preprocessing, like retina and cortex
vdWH Memtransistor [130]	SnS_2_/CIPS/h‐BN	10^6^	>10^4^ cycles, >10^4^ s	98%	Real‐time use of smart automobiles, high‐precision grasping, and target positioning with a robotic arm.
Ferroelectric transistor [131]	𝛼‐In_2_Se_3_	8 V	16 MLC states	99.5% (Dual feature)	NIR face recognition
Ferroelectric transistor [132]	2H 𝛼‐In_2_Se_3_	10^5^	>10^3^ cycles, 5000 s	95.55%	Image perception (‘U’ image) under optical pulses
Ferroelectric phototransistor [133]	graphene/ CIPS/hBN/MoTe_2_	10 V	>10^3^ s	92.4% (array size: 3 × 3)	Low‐light machine vision
Dual memtransistor [134]	MoS_2_/In_2_Se_3_/hBN (MIFeSS)	>10^3^	>10^3^ s, 20 MLC states	94.7%	Noise suppression, contrast enhancement

A key future direction involves the active engineering of 2D ferroelectric crystals to improve their intrinsic stability, enhance their polarization endurance, and broaden their optical absorption capabilities. The ongoing exploration of defect engineering, vacancy modulation, and controlled doping offers avenues to tune the internal electrostatic landscape of ferroelectric semiconductors. Such strategies not only influence polarization switching barriers but also modulate excitonic behavior, trap dynamics, and carrier mobilities, all of which directly impact the synaptic performances in optoelectronic devices. As demonstrated in recent studies, properly engineered defects can stabilize metastable ferroelectric phases and prolong retention times, resulting in the robust long‐term memory characteristics under repeated optical stimulation [81, 122]. In addition, Mixed‐dimensional vdW stacks enable a broad spectrum of functionalities, including wavelength‐selective detection, polarization‐dependent photocurrent generation, and programmable photoconductive states, further expanding the design space for multifunctional artificial vision hardware.

Beyond materials innovation, future opportunities increasingly depend on scalable fabrication and CMOS compatibility. High‐quality materials synthesis remains a significant challenge, as defects and grain boundaries can degrade the ferroelectric stability and device reliability. Additionally, controlling the uniformity and stability of ferroelectric polarization over large areas is critical for scalable device fabrication. Integration with existing semiconductor processes is another obstacle, requiring the development of a low‐temperature growth and transfer technique compatible with flexible substrates [49, 78]. Furthermore, understanding and engineering the long‐term endurance of ferroelectric states under repeated optical and electrical stimuli are crucial for reliable operation. Addressing these challenges necessitates a comprehensive approach involving material engineering, advanced fabrication techniques, and an in‐depth understanding of ferroelectric mechanisms at the nanoscale nodes [120]. Progress in back‐end‐of‐line (BEOL)‐compatible transfer process, low‐temperature growth methods, ALD‐based ferroelectric deposition, and polymer‐assisted lamination suggests that this barrier can be gradually overcome. bring the field closer to manufacturable integration.

Looking forward, the future landscape of artificial vision will likely be characterized by systems capable of performing decision‐making and pattern recognition directly during the sensing process. With the tunability offered by ferroelectric polarization and the enhanced photogating effects observed in 2D ferroelectric‐semiconductor heterostructures, vision sensors can encode environmental information in highly efficient analog formats. Such in‐sensor computational schemes reduce unnecessary data transmission and enable real‐time processing essential for applications such as robotics, autonomous navigation, human‐machine interaction, and wearable diagnostics. The ability to operate in ultra‐low voltages, coupled with mechanical deformity, further expands their applicability into artificial skins, and biomedical platforms, where intimate contact with soft tissues or conformal surfaces is necessary.

Finally, cross‐disciplinary efforts will be essential to convert these breakthroughs into viable technologies. Collaborative work across materials physics, neuromorphic computing, electrical engineering, and biomedical science will accelerate the translation of 2D ferroelectric optoelectronic systems into consumer electronics, medical devices, and intelligent robotic platforms. The long‐term vision includes the development of artificial retinas employing ferroelectric neuromorphic pixels, soft contact‐lens‐based imagers, fully autonomous drone vision modules, and energy‐efficient on‐chip processors integrated directly within the imaging hardware. In summary, 2D ferroelectric‐based optoelectronic vision sensors offer an exceptionally promising route toward intelligent artificial perception, and continued innovation in material engineering, scalable fabrication, device architecture optimization, and system‐level integration will ultimately enable vision technologies that surpass current limitations and bring artificial perception closure to the biological ideal.

## Author Contributions

Parthasarathi Pal: Writing – original draft, Methodology, Investigation, Formal Analysis, and Conceptualization, Writing – review & editing. Yeong‐Her Wang: Writing – review, Investigation, & editing, and Formal Analysis. Sanjay Kumar: Writing – review & editing, Conceptualization, Methodology, Investigation, Formal Analysis, Resources, and Supervision. Themis Prodromakis: Writing – review & editing, Formal Analysis, and Supervision.

## Conflicts of Interest

The authors declare that they have no known competing financial interests or personal relationships that could have appeared to influence the work reported in this paper.

## Data Availability

Data sharing not applicable to this article as no datasets were generated or analysed during the current study.
